# Fetal liver CD34^+^ contain human immune and endothelial progenitors and mediate solid tumor rejection in NOG mice

**DOI:** 10.1186/s13287-024-03756-7

**Published:** 2024-06-09

**Authors:** Teja Celhar, Xinyi Li, Yunqian Zhao, Hui Chien Tay, Andrea Lee, Hui Hua Liew, Edwin Kunxiang Shepherdson, Ravisankar Rajarethinam, Yiping Fan, Anselm Mak, Jerry Kok Yen Chan, Amit Singhal, Takeshi Takahashi

**Affiliations:** 1 Singapore Immunology Network (SIgN), Agency for Science, Technology and Research (A*STAR), 8A Biomedical Grove, Immunos #04-06, Singapore, 138648 Republic of Singapore; 2 Central Institute for Experimental Animals (CIEA), 3-25-12 Tonomachi, Kawasaki-ku, Kawasaki, Kanagawa 210-0821 Japan; 3 A*STAR Infectious Diseases Labs (A*STAR ID Labs), Agency for Science, Technology and Research (A*STAR), 8A Biomedical Grove, Immunos #05-13, Singapore, 138648 Republic of Singapore; 4 Department of Reproductive Medicine, KK Women’s and Children’s Hospital, Singapore, 229899 Republic of Singapore; 5 Institute of Molecular and Cell Biology (IMCB), Agency for Science, Technology and Research (A*STAR), 61 Biopolis Drive, Proteos, Singapore, 138673 Republic of Singapore; 6 Obstetrics and Gynaecology Academic Clinical Programme, Duke-NUS Medical School, Singapore, 169857 Republic of Singapore; 7 Experimental Fetal Medicine Group, Department of Obstetrics and Gynaecology, Yong Loo Lin School of Medicine, National University Health System, Singapore, 117597 Republic of Singapore; 8 Department of Medicine, Yong Loo Lin School of Medicine, National University of Singapore, Singapore, Singapore; 9 Division of Rheumatology, University Medicine Cluster, National University Health System, Singapore, Republic of Singapore; 10 Lee Kong Chian School of Medicine, Nanyang Technological University, Singapore, 636921 Republic of Singapore; 11 Interdisciplinary Life Sciences, Purdue University, West Lafayette, IN 47907 USA

**Keywords:** Humanized mice, HSPC, Fetal liver CD34, Cord blood CD34, Allogeneic tumor rejection, Graft-versus-tumor, LSEC, Sinusoidal dilatation, Endothelial disfunction

## Abstract

**Background:**

Transplantation of CD34^+^ hematopoietic stem and progenitor cells (HSPC) into immunodeficient mice is an established method to generate humanized mice harbouring a human immune system. Different sources and methods for CD34^+^ isolation have been employed by various research groups, resulting in customized models that are difficult to compare. A more detailed characterization of CD34^+^ isolates is needed for a better understanding of engraftable hematopoietic and potentially non-hematopoietic cells. Here we have performed a direct comparison of CD34^+^ isolated from cord blood (CB-CD34^+^) or fetal liver (FL-CD34^+^ and FL-CD34^+^CD14^−^) and their engraftment into immunocompromised NOD/Shi*-scid Il2rg*^*null*^ (NOG) mice.

**Methods:**

NOG mice were transplanted with either CB-CD34^+^, FL-CD34^+^ or FL-CD34^+^CD14^−^ to generate CB-NOG, FL-NOG and FL-CD14^−^-NOG, respectively. After 15–20 weeks, the mice were sacrificed and human immune cell reconstitution was assessed in blood and several organs. Liver sections were pathologically assessed upon Haematoxylin and Eosin staining. To assess the capability of allogenic tumor rejection in CB- vs. FL-reconstituted mice, animals were subcutaneously engrafted with an HLA-mismatched melanoma cell line. Tumor growth was assessed by calliper measurements and a Luminex-based assay was used to compare the cytokine/chemokine profiles.

**Results:**

We show that CB-CD34^+^ are a uniform population of HSPC that reconstitute NOG mice more rapidly than FL-CD34^+^ due to faster B cell development. However, upon long-term engraftment, FL-NOG display increased numbers of neutrophils, dendritic cells and macrophages in multiple tissues. In addition to HSPC, FL-CD34^+^ isolates contain non-hematopoietic CD14^+^ endothelial cells that enhance the engraftment of the human immune system in FL-NOG mice. We demonstrate that these CD14^+^CD34^+^ cells are capable of reconstituting Factor VIII-producing liver sinusoidal endothelial cells (LSEC) in FL-NOG. However, CD14^+^CD34^+^ also contribute to hepatic sinusoidal dilatation and immune cell infiltration, which may culminate in a graft-versus-host disease (GVHD) pathology upon long-term engraftment. Finally, using an HLA-A mismatched CDX melanoma model, we show that FL-NOG, but not CB-NOG, can mount a graft-versus-tumor (GVT) response resulting in tumor rejection.

**Conclusion:**

Our results highlight important phenotypical and functional differences between CB- and FL-NOG and reveal FL-NOG as a potential model to study hepatic sinusoidal dilatation and mechanisms of GVT.

**Supplementary Information:**

The online version contains supplementary material available at 10.1186/s13287-024-03756-7.

## Background

Humanized immune system (HIS) mouse models, which are immunodeficient mice engrafted with human hematopoietic stem and progenitor cells (HSPC) and/or tissues, are utilized to study various human immune cells and predict human response to drugs [1, 2]. NOD/Shi*-scid Il2rg*^*null*^ (NOG) [3] and NOD/LtSz-*scid Il2rg*^*null*^ (NSG) [4, 5] are the most common immunodeficient strains used for humanization. Both bare the severe combined immunodeficiency mutation Prkdc^*scid*^ on a nonobese diabetic (NOD) background and a mutation in the interleukin-2 (IL-2) receptor common gamma chain [5, 6]. Different methods have been employed for the generation of HIS-mice over the years, using different sources of human cells and tissues and various routes of injection [1, 2]. When transplanting exclusively HSPC, fetal liver (FL)-derived CD34^+^ (FL-CD34^+^) and cord blood (CB)-derived CD34^+^ (CB-CD34^+^) cells are injected intra-hepatically or via other routes into new-born mice [5, 7–14] or via the tail vein in adult mice [3, 7, 8, 15–22]. A single injection of human HSPC is technically simple and medium (10–20 mice) to large (> 30) cohorts of HIS mice can be generated from a single donor of CB or FL, respectively [23]. These HIS mice are generally healthy or develop GVHD after long-term engraftment, which allows a larger window for experimentation [7, 12, 24, 25].

FL is an attractive source of CD34^+^ HSPC, since > 1 × 10^8^ cells can be isolated from a single donor [26]. This enables the generation of large and/or multiple cohorts of HLA-defined humanized mice for tumor studies. When humanized mice are reconstituted with FL-CD34^+^ and used for cell line-derived xenograft (CDX) or patient-derived xenograft (PDX) engraftment, partial HLA matching is routinely applied [17, 27, 28]. The latter is to prevent tumor rejection by alloreactive T cells, also known as graft versus tumor (GVT) effect [29, 30]. When CB-CD34^+^ are used for reconstitution, HLA matching is not required for successful tumor growth and the HLA alleles are usually not mentioned or found irrelevant [18, 21, 22, 31–36]. This discrepancy suggests functional differences between FL- and CB-CD34^+^-reconstituted mice that might arise from distinct phenotypical characteristics. Importantly, as various research groups use different methods for mouse humanization in terms of CD34^+^ isolation, conditioning protocols, strain and age of mice, injection site and numbers of injected cells, a direct comparison of different CD34^+^ sources for humanization has yet to be accomplished.

Here, we have isolated CD34^+^ cells from CB and FL samples and found significant differences in their phenotype and reconstitution potential in adult NOG mice, referred to as CB-NOG and FL-NOG, respectively. Our results reveal that FL-CD34^+^ isolates contain a population of CD14^+^ endothelial cells that enhances the reconstitution of human immune cells in FL-NOG. These FL-CD34^+^CD14^+^ cells support the engraftment of human liver sinusoidal endothelial cells (LSECs) in FL-NOG but contribute to hepatic sinusoidal dilatation which can ultimately progress to a severe pathology that resembles graft-versus-host disease (GVHD). Finally, by employing an HLA-mismatched melanoma model, we show that graft-versus-tumor (GVT) can be induced in FL-NOG, but not CB-NOG. Therefore, the FL-NOG mice might serve as a novel model to study human LSEC disfunction and mechanisms of solid tumor rejection.

## Methods

### Mice

NOD/Shi*-scid Il2rg*^*null*^ (NOG) [3] and NOG-HLA-A24 transgenic (Tg) mice were obtained from The Central Institute for Experimental Animals (CIEA, Kawasaki, Japan). NOG-HLA-A24 Tg mice were established by backcrossing HLA-A24 Tg (C57/BL6) from Taconic Biosciences (Albany, NY, USA) onto the NOG background, as described previously for NOG-HLA-A2 Tg [37]. Mice were shipped from CIEA, Japan at 5–6 weeks of age to the Biological Resource Centre (BRC, A*STAR, Singapore) and let to acclimatize for a minimum of 7 days before any procedures were performed. Animals were housed in the BRC facility under specific pathogen-free conditions, on controlled 12 h light/dark cycles, and food and water ad libitum. Enrichment domes and nesting sheets were placed in all the cages. All animal experiments were performed in accordance with approved protocols by the Biological Resource Centre (BRC, A*STAR, Singapore) Institutional Animal Care and Use Committee (IACUC).

### Isolation of human HSCs

Human cord blood from full-term uncomplicated pregnancies and fetal liver tissue from clinically indicated termination of pregnancies of gestation age 14 to 21 weeks were collected at the KK Women’s and Children’s Hospital, Singapore. All the donations were performed in compliance with the SingHealth Centralised Institutional Review Board (CIRB) guidelines and written informed consent for tissue donation was obtained from all donors. Fetal liver tissue was aseptically collected, transported on ice and immediately immersed in cold phosphate buffered saline (PBS) with amphotericin B (5 µg/ml) (Fungizone), penicillin (200 U/ml) and streptomycin (200 µg/ml) (all from Gibco, Thermo Fisher Scientific, Walthan, MA, USA) for 10 min at 4 °C. A cell scraper was used to gently dissociate the tissue into a collagenase/dispase digestion medium (Liver Digest Media, Gibco, Cat# 17,703,034) supplemented with 4U/ml DNase I (Invitrogen, Thermo Fisher Scientific). The tissue was digested for 20 min at 37 °C, homogenized, filtered and the obtained suspension overlayed on Ficoll-paque Plus (Cytiva, Marlborough, MA, USA). Cord blood was diluted in a 1: 1 ratio with PBS and overlayed on Ficoll-paque Plus for density cell centrifugation. CD34^+^ HSPC were enriched from the mononuclear layer by two rounds of magnetic positive selection using the Ultrapure Human CD34 MicroBead Kit (Miltenyi Biotec, Bergisch Gladbach, Germany, Cat# 130-100-453). Purity of CD34^+^ HSPC cells was assessed by flow cytometry (Additional file 1: Fig. S1 and Additional File 2: Table S1), and cells were cryopreserved in Bambanker (GC Lymphotec, Tokyo, Japan). The viability of freshly isolated CB-CD34^+^ was higher compared to FL-CD34^+^ (assessed by DAPI staining; Additional File 1: Figure S1A). Upon thawing, the cells were re-counted twice, and the average trypan-blue negative cell counts were used to calculate the cell dose for transplantation.

### Flow cytometric analysis of CB- and FL-CD34^+^

CD34^+^ were thawed, washed with PBS, stained with the LIVE/DEAD™ Fixable Blue Dead Cell Stain Kit (Invitrogen, Thermo Fisher Scientific) and Fc receptors blocked with TruStain FcX™ (BioLegend, San Diego, CA, USA) for 15 min. Primary antibody staining was performed at 4 °C for 30 min with antibody panels listed in Additional File 2: Table S2. For VE-cadherin (CD144) intracellular staining the cells were permeabilized and fixed using the BD Cytofix/Cytoperm (BD Biosciences, Franklin Lakes, NJ, USA) kit following the manufacturer’s instruction. Cells were resuspended in staining buffer and run on BD LSR5 or BD Symphony A5.2 with the FACSDiva™ software (all from BD Biosciences). Analysis was performed using Flowjo Software (v. 10.8.1) (Flowjo, LLC, Ashland, OR, USA).

### FL-CD34^+^ sorting and cytospin

FL-CD34^+^ were thawed, stained for Fluorescently Activated Cell Sorting and sorted on FACSAria (BD Biosciences) to deplete CD14^+^ cells as shown in Additional file 1: Fig. S2. For morphology assessment, the sorted CD14^+^ and CD14^−^ fractions were cytospined onto slides using a Shandon Cytospin 4 centrifuge (Thermo Fischer Scientific). Eosin and haematoxylin-stained slides were mounted with Entellan rapid mounting medium (Merck, Darmstadt, Germany), imaged at 100x magnification using a BX43 light microscope (Olympus, Tokyo, Japan) and photomicrographs captured by an Olympus DP21 camera.

### Human cell transplantation

6 weeks old male NOG mice were sub-lethally irradiated with 1.2 Gy (RS 2000 X-Ray irradiator; Rad Source Technologies Inc., Buford, GA, USA) and injected with 5 × 10^4^ live human CB-CD34^+^, FL-CD34^+^ or an equivalent number of live FL-CD34^+^CD14^−^ in PBS through the tail vein (i.v.). Three independent cohorts of mice were generated, with CD34^+^ isolated from distinct CB (*n* = 3) and FL (*n* = 3) donors. The FL-CD34^+^CD14^−^ were sorted from one of the FL donors and engrafted alongside unsorted cells within the same cohort. Detailed cohort, mouse and donor information can be found in Additional File 2: Table S3. No specific randomisation method was used for mouse or cage allocation. Mice were monitored weekly for any adverse effects, including weight loss, pallor, hair loss and weakness. As the experiment was designed to directly compare two groups, no exclusion criteria were set *a priory*, however three experimental animals were excluded due to unexpected death (two accidental and one unspecified; details in Additional File 2: Table S3).

For CDX experiments, 6–8 weeks old male and female NOG and NOG-HLA-A24 Tg mice were sub-lethally irradiated with 1.2 Gy and injected i.v. with 5 × 10^4^ CB-CD34^+^ or 7.5 × 10^4^ FL-CD34^+^ in PBS. Four independent cohorts of mice were reconstituted with one CB-CD34^+^ and two FL-CD34^+^ donors, with no specific randomisation method for mouse or cage allocation. Detailed mouse records are found in Additional File 2: Table S4.

The various group allocation was known to everyone at different stages of the experiment, except to the pathologist to whom the groups were revealed only after the pathology assessment and scoring.

### Humanized mouse tissue isolation and processing

Graduated capillaries with heparin were used to collect 100 µl peripheral blood from the retro-orbital venous plexus of mice anesthetized by isoflurane inhalation at 4, 8, 12, 15 and 20 weeks post-CD34 + transplantation. Red blood cells were lysed with Pharm Lyse (BD Biosciences). For endpoint analyses mice were euthanized by CO2 inhalation at 15 or 20 weeks post transplantation. Bone marrow (BM) samples were collected by flushing one femur with 1 ml of cold PBS. Spleens were crushed with a syringe plunger, digested with 0.2 mg/ml collagenase IV (Sigma-Aldrich, Burlington MA, USA) supplemented with 0.03 mg/ml DNAse I (Roche Diagnostics GmbH, Mannheim, Germany) for 30 min at 37 °C. Lungs were cut into smaller pieces with scissors, digested with 1.4 mg/mL Collagenase A (Merck) and 0.03 mg/ml DNase I for 1 h at 37 °C. Livers were cut into smaller pieces and digested with 0.2 mg/ml collagenase IV with 0.05 mg/ml DNAse for 45 min at 37 °C. Liver single cell suspensions were resuspended in 40% isotonic Percoll PLUS (Sigma-Aldrich) and centrifuged to remove the top layer of fat and tissue cells. ACK lysis buffer (Gibco, Thermo Fisher Scientific) was used to lyse red blood cells.

### Flow cytometric analysis of blood and tissue-infiltrating immune cells

For reconstitution kinetics, blood cells were stained with the antibodies listed in Additional file 2: Table S5, acquired on FACSCanto II (BD Biosciences) and gated as shown in Additional file 1: Fig. S3. Antibody panels for endpoint analyses of blood, bone marrow, spleen and lungs can be found in Additional file 2: Table S6, with gating strategies in Additional file 1: Fig. S4. Non-specific staining was blocked with human (TruStain FcX™, BioLegend) and mouse (2.4G2 hybridoma supernatant) Fc block for 15 min. Extracellular markers were stained at 4 °C for 30 min. For intracellular staining, BM, spleen and liver samples were permeabilized and fixed using the BD Cytofix/Cytoperm kit (BD Biosciences) following the manufacturer’s instruction. Lung cells were fixed in 2% PFA. Live/dead exclusion was achieved by resuspending in DAPI (Invitrogen, Thermo Fisher Scientific) for unfixed blood cells, while all fixed samples were pre-stained with the LIVE/DEAD™ Fixable Blue Dead Cell Stain Kit (Invitrogen, Thermo Fisher Scientific). The samples were acquired on a LSR5 (BD Biosciences) with FACSDiva™ software (BD Biosciences). Analysis was performed using Flowjo Software (v. 10.8.1).

### Cell line-derived xenograft (CDX) tumor model

After confirming the reconstitution of T cells (14–16 weeks post HSPC transplantation), the humanized mice were first injected intraperitoneally (i.p.) with 3 doses of 10 µg Flt3L-Fc (BioXcell, Lebanon, NH, USA) given every 2 days to induce the generation of DCs [38]. This was followed by i.p. immunization with 1 × 10^6^ non-viable A375 melanoma tumor cells treated with mitomycin-C (Sigma-Aldrich). After 7 days of priming, mice anesthetized with isoflurane were subcutaneously injected with 1 × 10^6^ viable A375 cells in the right flank and tumor growth was measured twice a week using calipers (schematic of the experiment in Additional File 1, Fig. S5). Animals were monitored bi-weekly for tumor necrosis and adverse effects such anemia or skin GVHD (pallor, weight loss, weakness, hair loss) and euthanized on humane grounds by CO2 inhalation in case of necrotic tumor or severe symptoms. As all the outcomes were important for the study, including the development of any adverse reactions, no experimental animals were excluded from the study. For records of tumor rejection and adverse reaction frequency see Additional File 2: Table S4. Animals not displaying adverse effects were euthanized by CO2 inhalation at the endpoints indicated in Additional File 2: Table S4.

### Histology

Tissues were harvested and fixed in 10% NBF for about 48 h at RT. Fixed tissues were processed in ascending grades of alcohol, cleared in Xylene, infiltrated with paraffin wax and cut at 5 μm thickness. The tissue sections were brought to water and stained with Haematoxylin and Eosin (H&E). Histo-pathological assessment was performed by pathologist in a blind fashion using Olympus BX51 upright microscope and representative photomicrographs were captured with an Olympus DP71 digital colour camera and cellSens Imaging software (Olympus Life Science, Tokyo, Japan).

### Multiplex microbead-based immunoassay

MILLIPLEX MAP Human Cytokine/Chemokine Magnetic Bead Panel – Premixed 41 Plex- Immunology Multiplex Assay (Cat#HCYTMAG-60 K-PX41, Merck/Millipore, Darmstadt, Germany) was used to measure serum cytokines. Samples were acquired on the FLEXMAP® 3D (Luminex, Thermo Fischer Scientific) using xPONENT® 4.0 (Luminex) software and data analysed with Bio-Plex ManagerTM (v. 6.1.1; Bio-Rad, Hercules, CA, USA).

### ELISAs

ALT was measured in plasma with the Mouse ALT Elisa Kit (Fine Biotech Co., Ltd, Wuhan, China) according to the manufacturer’s instructions. Factor VIII was measured in the plasma with the Human FVIII ELISA kit (Abcam, Cambridge, UK) according to the manufacturer’s instructions, where human plasma was diluted 1:50 and humanized mouse plasma 1:2.

### HLA typing

DNA was extracted from the CD34^−^ fraction of CB and FL samples using the PureLink® Genomic DNA Kit (Invitrogen, Thermo Fisher Scientific). Sequence based typing (HLA Laboratory, Blood Services Group, Health Sciences Authority, Singapore) was used to determine the genotype of the HLA-A alleles.

### Statistical methods

GraphPad Prism (v 9.4.1, GraphPad Software, La Jolla, CA, USA) was used to prepare graphs and perform statistical comparisons. The Kolmogorov-Smirnov test was used for normality calculations. Two groups without normal distribution were compared with the Mann-Whitney test and, where indicated, corrected for multiple comparisons using the Holm-Sidak method. Three groups were compared with an ordinary one-way ANOVA with post-hoc Tukey if normally distributed or alternatively, with a Kruskal-Wallis test with post-hoc Dunn’s. All correlations were calculated using Spearman correlation, as the pathology scoring data was not normally distributed. P values < 0.05 were considered statistically significant.

## Results

### CD34^+^ cells isolated from fetal liver have a distinct phenotype compared to cord blood- CD34^+^

**Fig. 1:**
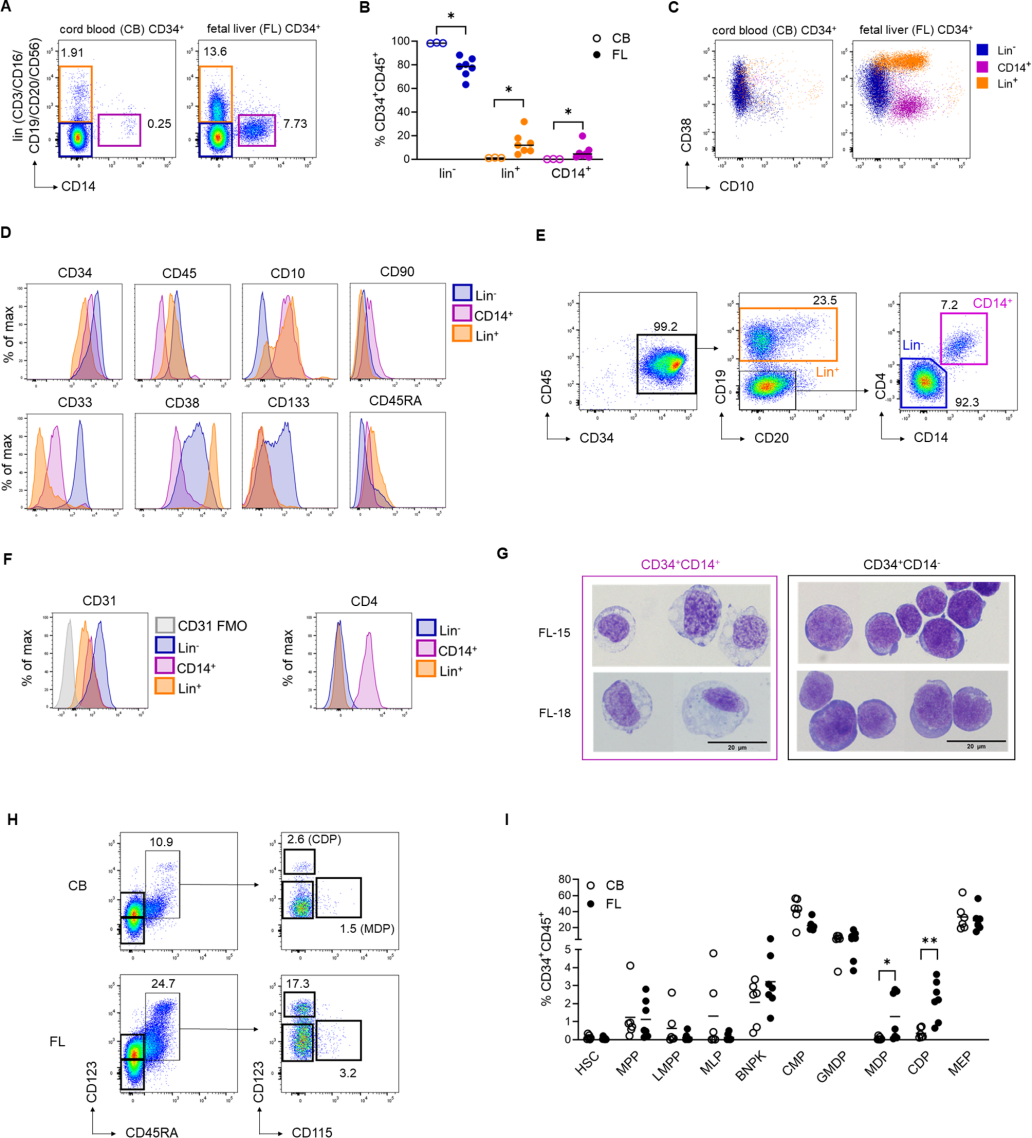
FL-CD34^+^ are a heterogeneous mixture of hematopoietic and non-hematopoietic cells. (**A**) Representative flow cytometry plots of CB- and FL-CD34^+^ (gated on singlet, live, CD34^+^CD45^+^) showing percentages of lin^−^CD14^−^ (indigo), lin^+^ (orange) and CD14^+^ (magenta) with (**B**) cumulative frequencies of each population. (**C**) Representative flow cytometry dot plots showing expression of CD38 vs. CD10. (**D**) Representative histograms showing the expression levels (median fluorescence level) of the indicated markers on the three populations identified within FL-CD34^+^. (**E**) Gating strategy and (**F**) corresponding histograms showing expression levels of CD31 and CD4 on the indicated populations. (**G**) Giemsa staining of sorted FL-CD34^+^CD14^−^ and CD34^+^CD14^+^ from two representative donors. (**H**) Representative flow cytometry plots of CB- and FL-CD34^+^ (gated on singlet, live, CD34^+^CD45^+^lin^−^CD14^−^CD38^+^CD10^−^; for complete gating strategy see Additional File 1, Fig. S6E) showing frequencies of CDP and MDP and (**I**) cumulative data of progenitor frequencies contained within CD34^+^lin^−^CD14^−^, calculated as % of CD34^+^CD45^+^. Each symbol represents an individual donor, horizontal lines indicate mean. Statistical comparisons by two-tailed Mann-Whitney, *p < 0.05, **p < 0.01. Abbreviations: FMO: fluorescence minus one; HSC: hematopoietic stem cells; MPP: multipotent progenitor; LMPP: lymphoid-primed multi-potent progenitor; LMPP: lymphoid-primed multi-potent progenitor; MLP: multi-lymphoid progenitor; BNPK: B/NK progenitor; CMP: common DC progenitor; GMDP: granulocyte-monocyte-DC progenitor; MDP: monocyte-DC progenitor; CDP: common DC progenitor; MEP: megakaryocytic-erythroid progenitor

CD34^+^ cells isolated from CB and FL were evaluated for purity and phenotypical characteristics. The frequency of CD34^+^ from both sources was similar (> 95%) (Additional File 1: Fig S1A, B), however a considerable proportion of FL-CD34^+^ was positive for lineage (lin) markers CD3/CD14/CD16/CD19/CD20/CD56 (Fig. 1A, B, Additional File 1: Fig. S1A). This lowered the fraction of CD34^+^lin^−^ in FL-CD34^+^ to a median of 66.5% compared to 94.8% in CB-CD34^+^ (Additional File 1: Fig. S1C) and prompted us to perform in-depth phenotyping of the CD34^+^ isolates. By using an extended flow cytometric panel (Additional File 2: Table S6), we confirmed that CB-CD34^+^ demonstrated a relatively uniform population of CD34^+^lin^−^CD14^−^ (lin^−^) cells (Fig. 1A-C). Contrary, FL-CD34^+^ consist of at least three distinct populations: lin^−^ (indigo), lin^+^ (orange) and CD14^+^ (magenta) (Fig. 1A-C). The lin^+^ cells are predominately CD19^+^ B cell precursors and express CD45RA, CD10 and high levels of CD38^+^ (Fig. 1C-E and Additional File 1: Fig. S6A-B). CD14^+^ express CD10 and CD90, low levels of CD45 and are low/negative for the stem cell marker CD133 (Fig. 1C, D and Additional File 1: Fig. S6A); a phenotype characteristic of liver sinusoidal endothelial cells (LSECs) [39, 40]. CD14^+^ cells were positive for the endothelial markers VE-cadherin (CD144) and CD31, however the latter was not specific as it was highly expressed also on CD34^+^lin^−^ cells (Fig. 1E, F and Additional File 1: Fig. S6B-D). A recent report by Popescu et al. described a CD34^+^CD14^+^CD4^+^ endothelial population within the fetal liver [41] and we confirmed CD4 as a highly specific marker for these cells (Fig. 1E-F and Additional File1: Fig.S6D). We verified the endothelial morphology by Giemsa staining, showing a characteristic vacuolated and blebbing cytoplasm [41, 42] (Fig. 1G, left panel). In contrast, the CD34^+^CD14^−^ fraction presented with a high nuclei-to-cytoplasm ratio, typical of blast/stem cells [42] (Fig. 1G, right panel).

Lastly, we compared the progenitor populations contained within the CD34^+^lin^−^CD14^−^ [43]. By applying a Fluorescence Minus One (FMO) control for CD38, we determined that the majority of CD34^+^ isolated from both sources are CD38^+^ late progenitors (Additional File 1: Fig. S6E). Overall, the progenitor profile of CB- and FL-CD34^+^lin^−^CD14^−^ cells was similar, except for frequencies of common dendritic cell (DC) progenitors (CDP) and monocyte-DC progenitors (MDP), which were significantly higher among FL-CD34^+^lin^−^CD14^−^ (Fig. 1H, I). In summary, CB-CD34^+^ consist predominately of lin^−^ HSPC, while FL-CD34^+^ cells additionally contain B cell lineage-committed lin^+^CD38^high^ progenitors and non-hematopoietic CD14^+^ endothelial cells.

### FL-NOG engraft human endothelial and immune cells

**Fig. 2:**
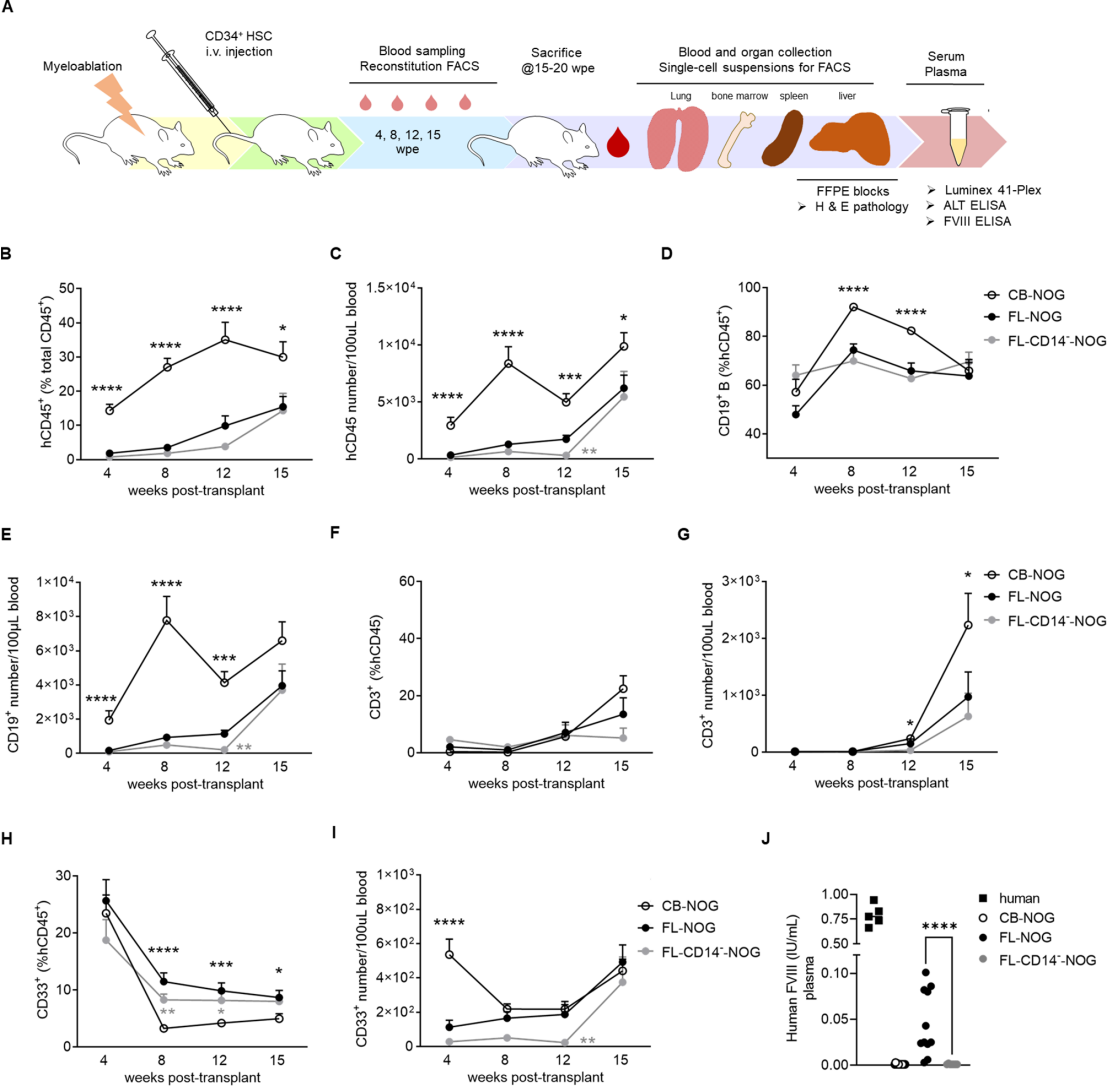
Reconstitution kinetics is faster in CB- compared to FL-NOG. (**A**) Schematic of the experimental design. Sublethally irradiated NOG mice were injected with 5 × 10^4^ CB-CD34^+^ or FL-CD34^+^ or equivalent number of FL-CD34^+^CD14^−^. Mice were sacrificed at either 15 or 20 wpe and blood, tissues, serum and plasma were collected for analysis. Immune cell reconstitution in the peripheral blood was measured by flow cytometry at 4, 8, 12 and 15 weeks post-transplant. Diagrams of mean with standard error of the mean (SEM) showing (**B**) percentages and (**C**) numbers of human CD45^+^ (hCD45); (**D**) percentages of CD19^+^ B within hCD45 and (**E**) their absolute numbers; (**F**) percentages CD3^+^ T within hCD45 and (**G**) their absolute numbers; (**H**) percentages of CD33^+^ myeloid cells and (**I**) and their absolute numbers in peripheral blood at indicated time points. (**J**) Human Factor VIII (FVIII) measured by ELISA in human plasma and plasma of indicated humanized mice at endpoint. For B-I, mean + SEM is shown for *n* = 15 (CB-NOG), *n* = 13 (FL-NOG) and *n* = 7 (FL-CD14^−^- NOG). Statistical comparisons were calculated by the Multiple Mann-Whitney tests corrected for multiple comparisons using the Holm-Sidak method. * *p* < 0.05, ** *p* < 0.01, *** *p* < 0.001, **** *p* < 0.0001; shown in black for CB vs. FL and in gray for FL vs. FLCD14^−^ comparisons. For J, individual mice are shown for each group (*n* = 5 for human plasma, *n* = 14 for CB-NOG, *n* = 11 for FL-NOG and *n* = 7 for FL-CD14^−^-NOG. The statistical comparison was calculated by the Mann-Whitney test, **** *p* < 0.0001

As the composition of FL-CD34^+^ significantly differs from CB-CD34^+^ (Fig. 1), we predict that transplanting equal numbers of FL-CD34^+^ and CB-CD34^+^ in NOG might lead to differential reconstitution of immune cells. To evaluate this, we performed a comprehensive side-by-side longitudinal analysis of CB-CD34^+^ and FL-CD34^+^-transplanted NOG mice (CB-NOG and FL-NOG; respectively) (Fig. 2A). The CD14^+^ endothelial cell population contained within FL-CD34^+^ might affect the reconstitution of the hematopoietic stem cells [10, 44] and/or contain engraftable CD14^+^CD34^+^ LSECs or their precursors [39, 45]. Hence, we also transplanted NOG with CD14^+^-depleted FL-CD34^+^ (FL-CD14^−^-NOG) (Additional File 1: Fig. S2) alongside total FL-CD34^+^.

The kinetics of human immune CD45 (hCD45) cell reconstitution in the blood of CB-NOG was faster than in FL-NOG by percentages and absolute numbers (Fig. 2B, C). CD14^+^ depletion additionally delayed hCD45 reconstitution in FL-CD14^−^-NOG (Fig. 2C). CB-NOG displayed increased reconstitution of hCD19^+^ B cells compared to FL-NOG within the first 12 weeks post-transplant (wpt; Fig. 2D, E). Similarly, CD3^+^ T cell numbers were higher in CB-NOG at 12 and 15 wpt (Fig. 2F, G). On the other hand, FL-NOG reconstituted higher frequencies of CD33^+^ myeloid cells at 8, 12 and 15 wpt (Fig. 2H). Absolute numbers of CD33^+^ and CD19^+^, but not CD3^+^, were lower at 12 wpt in FL-CD14^−^-NOG in comparison to FL-NOG (Fig. 2E, G, I). Overall, transplantation with FL-CD34^+^ results in a slower reconstitution of the human immune system compared to CB-CD34^+^, which is further delayed by the depletion of CD14^+^ endothelial cells. However, FL-NOG reconstitute a larger proportion of CD33^+^ myeloid cells, in accordance with a higher frequency of MDP and CDP within FL-CD34^+^ (Fig. 1H, I).

LSECs are among the main producers of Factor VIII (FVIII) [46] and human FVIII can be detected upon successful LSEC engraftment in humanized mice [39, 45]. Thus, to evaluate human endothelial cell engraftment, we measured human FVIII in the plasma of humanized NOG and compared it to human samples. Our data clearly demonstrates that the engraftable FVIII-producing LSECs are contained within FL-CD34^+^ and are completely removed by CD14^+^-depletion (Fig. 2J).

### Differences in long-term multi-lineage reconstitution of the human immune system in CB- and FL-NOG mice

**Fig. 3:**
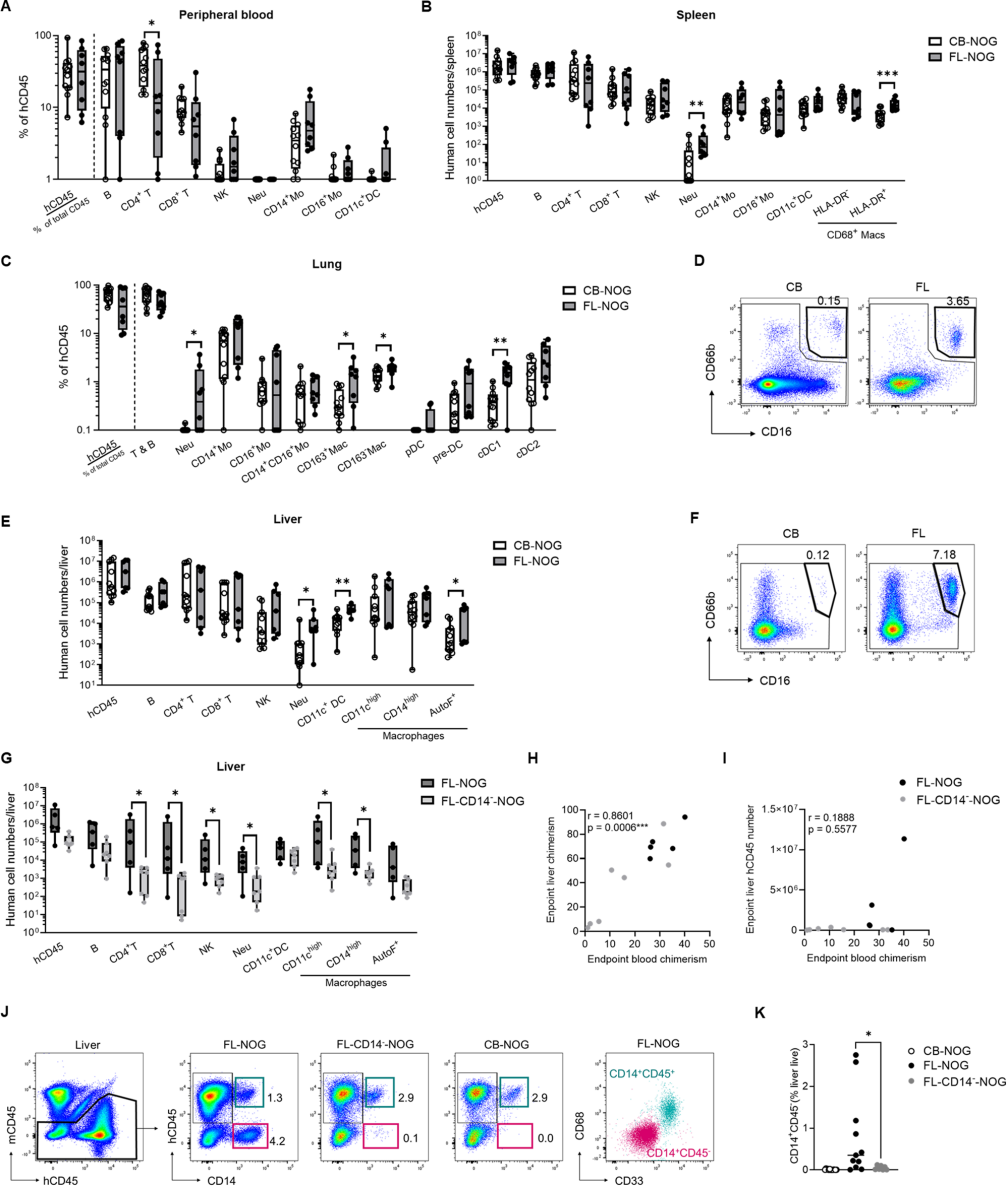
FL-NOG reconstitute more neutrophils and certain myeloid populations within tissues which is supported by FL-CD34^+^CD14^+^ cells. Mice were euthanized 20 weeks after transplantation (*n* = 8 for FL-NOG and *n* = 12 for CB-NOG), and bone marrow BM (1 femur), peripheral blood, spleen, lung and liver were collected and analysed by flow cytometry. (**A**) Frequencies of human immune cells in peripheral blood and (**B**) numbers in spleen. (**C**) Percentages of human immune cells in lungs with (**D**) representative FACS plots showing CD16^+^CD66b^+^ neutrophils. (**E**) Numbers of human immune cells in liver with (**F**) representative FACS plots showing CD16^+^CD66b^+^ neutrophils. (**G**) Cell numbers from livers harvested at 15–20 weeks after transplantation from mice transplanted with FL-CD34^+^ cells or an equivalent number of FL-CD34^+^CD14^−^ from the same donor (*n* = 5 for FL-NOG and *n* = 7 for FL-CD14^−^-NOG). Box and whiskers plots showing all points from min to max. Spearman correlations of endpoint blood hCD45 chimerism with (**H**) hCD45 liver chimerism at endpoint and (**I**) total hCD45 number per liver at endpoint with indicated Spearman’s r and p value. (**J**) Gating strategy and representative flow cytometry plots showing liver engraftment of CD14^+^CD45^+^ macrophages and CD14^+^CD45^−^ endothelial cells in the livers of indicated humanized mice with (**K**) cumulative data calculated as percentage of live singlets. Each symbol represents a single mouse. Statistical comparisons were calculated by Mann-Whitney tests, *p < 0.05, **p < 0.01, ***p < 0.001, ****p < 0.0001

Upon assessing the chimerism in the peripheral blood, we next evaluated the differential multilineage reconstitution of human immune cells in various tissues at endpoint (Additional File 2: Table S6, gating strategies in Additional File 1: Fig. S4). At 20 wpt the frequency of T cells, specifically CD4^+^ T, continued to be higher in the blood of CB-NOG compared to FL-NOG (Fig. 3A). The frequencies and numbers of all other analysed immune cells were comparable (Fig. 3A and Additional File 1: Fig. S7A). In the bone marrow (BM) we detected human neutrophils at various stages of development (Additional File 1: Fig. S4B) and the proportion of mature CD66b^+^CD16^high^ neutrophils was significantly higher in FL-NOG compared to CB-NOG (Additional File 1: Fig. S7B). Except for two mice, no mature human neutrophils were found in the peripheral blood of humanized NOG mice irrespective of the CD34^+^ source (Fig. 3A and Additional File 1: Fig. S7A). Though, almost all animals had detectable neutrophils in the spleen, lung and liver and FL-NOG had significantly more neutrophils within all the analysed organs (Fig. 3B-F and Additional File 1: Fig. S7C, D). Additionally, FL-NOG spleens contained more HLA-DR^+^CD68^+^ macrophages (Fig. 3B), and FL-NOG livers had more abundant CD11c^+^ dendritic cells (DC) and auto-fluorescent macrophages (Fig. 3E). Several myeloid populations (cDC1, CD163^+^ and CD163^−^ macrophages) were also more frequent in the lungs of FL-NOG (Fig. 3C).

Depleting CD14^+^ cells prior FL-CD34^+^ transplantation significantly decreased the numbers of human CD4^+^T, CD8^+^T, NK cells, neutrophils and macrophages within the liver (Fig. 3G). We observed a strong linear correlation between blood and liver hCD45 chimerism in FL- and FL-CD14^−^-NOG (Fig. 3H). However, total liver hCD45^+^ numbers did not increase along with blood chimerism in FL-CD14^−^-NOG mice (Fig. 3I), suggesting endothelial CD14^+^ cells might be important for immune cell recruitment or homing within the liver. To verify the engraftment of CD14^+^CD45^−^ LSEC endothelial cells in the livers of FL-NOG mice, we have checked the presence of CD14-expressing cells within the mouse CD45 negative (mCD45^−^) fraction (Fig. 3J). We specifically detected CD45^−^CD14^+^ cells in the livers of FL-NOG which were almost depleted in FL-CD14^−^- NOG and absent in CB-NOG livers (Fig. 3J, K). These cells were clearly distinguishable from CD14^+^ macrophages, which expressed CD33 and CD68 (Fig. 3J, far right panel). Additionally, no such cells were detected in the lung or spleen (Additional File 1: Fig. S7F, G), suggesting specific homing of these cells within the liver tissue.

To summarize, the increased reconstitution of several myeloid populations in FL-NOG compared to CB-NOG was consistent with an increased frequency of myeloid progenitors contained within FL-CD34^+^. Additionally, our data suggests that the endothelial FL-CD34^+^CD14^+^ may support the recruitment of FL-CD34^+^CD14^−^ reconstituted immune cells to the liver through direct engraftment within the hepatic tissue.

### FL-CD34^+^-transplanted NOG mice develop a distinct GVHD liver pathology

**Fig. 4:**
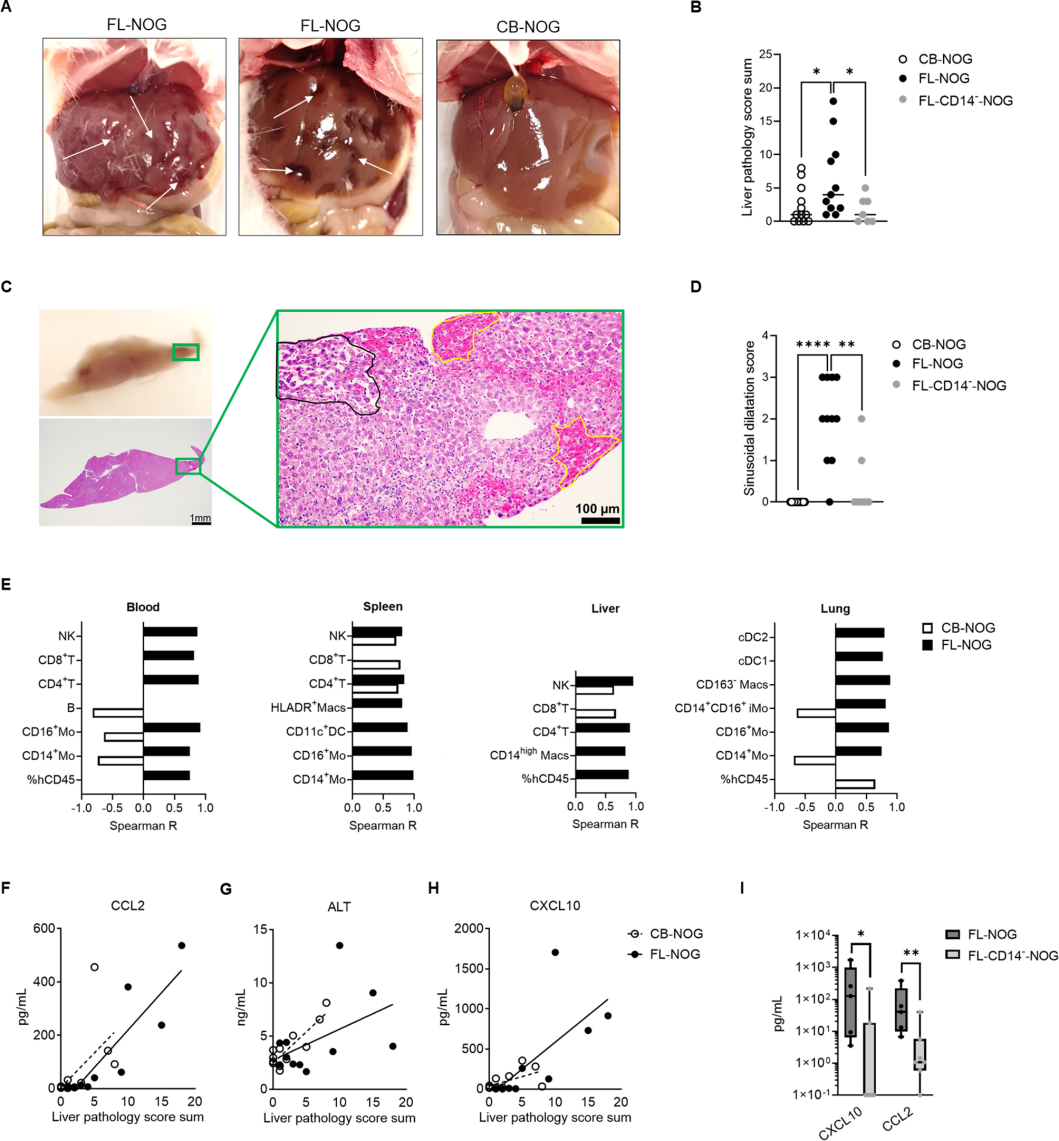
Long-term reconstituted FL-NOG develop a distinct spotted liver pathology associated with accumulation of lymphoid and myeloid cells. (**A**) Gross pathology of livers from FL-NOG mice at showing a spotted appearance and a representative CB-NOG liver with (**B**) cumulative liver pathology scores at endpoint. (**C**) Photo of a representative formalin-fixed paraffin embedded liver block with the corresponding H&E-stained section and magnified region of a dark spot showing an area of sinusoidal dilatation (outlined in black) and multifocal haemorrhages outlined in yellow. (**D**) SD pathology scores at endpoint. (**E**) Histograms showing hCD45 chimerism and cell numbers with significant (*p* < 0.05) positive (Spearman *R* > 0.0) or negative (Spearman *R* < 0.0) correlation with cumulative liver pathology scores in CB- and FL-NOG (exact R and p values in Additional File 2: Table S7). (**F**) Scatter plots and simple linear regression lines showing correlation among cumulative pathology score and serum levels of alanine transaminase (ALT), CCL2 and CXCL10 (exact R and p values in Additional File 2: Table S8). (**G**) CXCL10 and CCL2 in the serum of FL- and FL-CD14^−^-NOG at endpoint. Each symbol represents a single mouse; box and whiskers plots showing all points from min to max, statistical comparisons among two groups by Mann-Whitney tests, *p < 0.05, **p < 0.01, ****p < 0.001. Details of experimental mice used for each graph in Additional File 2: Table S2

Long-term reconstitution of the human immune system in immunodeficient mice can lead to xenogeneic graft-versus-host-disease (GVHD) [9, 12, 47, 48]. In our experiments all mice survived until endpoint without weight loss (not shown), however 3 out of 11 (27%) FL-NOG mice developed visible dark spots on the liver (Fig. 4A) and 2 out of 12 (16.7%) CB-NOG developed facial alopecia (Additional File 1: Fig. S8A). The cumulative liver pathology score was most severe in FL-NOG (Fig. 4B and Additional File 1: Fig. S8B). Histopathological analysis identified that the dark spotting of the liver was due to multifocal haemorrhages, which were associated with multifocal hepatic necrosis (Fig. 4C, Additional File 1: Fig. S8B). Sinusoidal dilatation (SD) was present in 10 out of 11 (91%) FL-NOG livers, even when no other pathology was present (Fig. 4D; Additional File 1: Fig. S8B, C). FL-CD14^−^-NOG had a significantly reduced incidence of these phenotypes (Fig. 4B, D), suggesting that human CD14^+^CD45^−^ LSEC engraftment contributes to the liver pathology in FL-NOG.

To identify specific cell types and mediators associated with the overall liver pathological score, we performed Spearman correlation analyses (Additional File 2: Tables S7, S8). Liver pathology in FL-NOG correlated positively with both lymphoid and myeloid cell numbers across multiple tissues (Fig. 4E). Whereas, in CB-NOG, it positively correlated only with human lymphoid cell numbers, specifically with CD4^+^ T, CD8^+^T and NK in spleen and/or liver (Fig. 4E). Multiple cytokines and chemokines were associated with the pathology score in both CB- and FL-NOG, including CCL2/MCP-1 (Fig. 4F, Additional File 1: Fig. S8D, Additional File 2: Table S8). Increased levels of alanine transaminase (ALT) positively correlated with liver inflammation exclusively in CB-NOG (Fig. 4G), while CXCL10 was among the cytokines important for driving the pathology solely in FL-NOG (Fig. 4H, Additional File 1: Fig. S8D, Additional File 2: Table S8). LSEC have been shown to produce CXCL10 and CCL2 under inflammatory conditions upon acute liver injury [49]. Depletion of CD14^+^ LSEC-reconstituting cells prior transplantation of FL-CD34^+^, significantly decreased serum levels of human CXCL10 and CCL2 (Fig. 4I).

In summary, long-term reconstitution with CB- and FL-CD34^+^ can lead to distinct GVHD-associated pathological changes in NOG mice. In FL-NOG we detected a previously undescribed spotted liver pathology characterized by sinusoidal dilatation, systemic increase in lymphoid and myeloid cell numbers and secretion of CXCL10. This GVHD-like phenotype was found to be due to presence of endothelial CD14^+^ cells among FL-CD34^+^.

### Allogeneic solid tumors are rejected in FL-CD34^+^-reconstituted mice

**Fig. 5:**
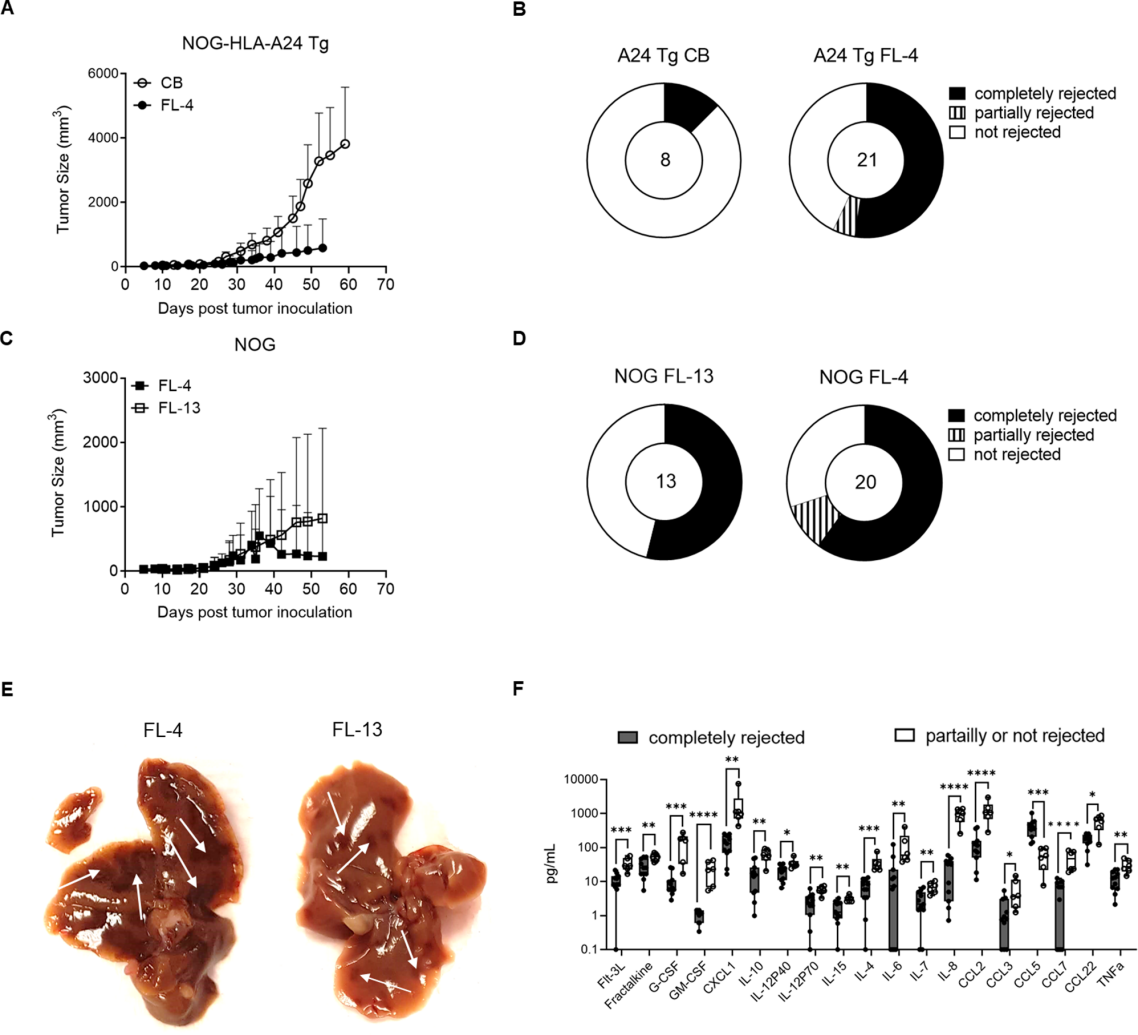
FL-NOG reject HLA-A mismatched tumors. A375 melanoma CDX was engrafted in the right flank of mice reconstituted with HLA-A mismatched CD34^+^-HSPC. (**A**) Tumor growth curves of A375 in NOG-HLA-A24 Tg (A24 Tg) mice reconstituted with CB- or FL-CD34^+^ with (**B**) corresponding donut charts showing the proportion of mice completely rejecting the tumors. (**C**) Growth curves of A375 in NOG mice reconstituted with two FL donors with (**D**) corresponding donut-charts showing the proportion of mice completely or partially rejecting the tumors. Growth curves are plotted as mean + SD, total n for each condition is indicated in the centre of the donut charts. (**E**) Photographs of gross pathological alterations of the liver; white arrows pointing to light to dark brown coloured irregular lesions noticed on the visceral surface of two representative livers from mice in C-D. (**F**) Serum cytokines and chemokines in representative mice from C-D that completely rejected the tumors (*n* = 11) vs. the rest of the mice (*n* = 6). Box and whiskers plots showing all points from min to max, statistical comparisons among two groups by Mann-Whitney tests, *p < 0.05, **p < 0.01, ***p < 0.001,****p < 0.001

To assess the functionality of CB- and FL-CD34^+^ reconstituted mice, we next tested their ability to mount an allogeneic anti-tumor response using an A375 melanoma CDX model (Additional File 1: Fig. S5, HLA-A alleles in Additional File 2: Table S9). In conventional NOG mice, the human T cells are selected and educated in the mouse thymus on mouse MHC [50]. To stimulate the development of human HLA-A*24 restricted T cells from A*24:02 donor HSPCs, we first opted for NOG mice that express human HLA-A*24:02 (NOG-HLA-A24 Tg or A24 Tg in short). Except for one mouse, A375 CDX were not rejected in mice reconstituted with HLA-A mismatched CB-CD34^+^ (Fig. 5A, B). In contrast, A375 CDX did not grow well in mice reconstituted with mismatched FL-CD34^+^ and after being initially palpable, tumors were completely rejected in > 50% of the mice (Fig. 5B). To test whether the HLA-A Tg was necessary for the observed rejection, we repeated the same experiment in conventional NOG mice transplanted with two FL donors, FL-4 and FL-13. In both cases, > 50% of mice completely rejected the tumors (Fig. 5C, D). In these experiments, we transplanted the mice with 7.5 × 10^5^ FL-CD34^+^ to achieve similar reconstitution levels to CB-CD34^+^ (Additional File 1: Fig. S9A). The higher cell dose resulted in a high incidence of the spotted liver phenotype, which was 50–100% depending on the cohort. Donor FL-4 induced a more severe pathology compared to FL-13 by visual inspection with corresponding higher levels of serum cytokine/chemokine (Fig. 5E, Additional File 1: Fig. S9B). Additionally, FL-4 reconstituted NOG mice that did not completely reject the tumors, were significantly more prone to develop weakness and pallor leading to premature death or requiring euthanasia (Additional File 1: Fig. S9C-E). Failure of complete rejection resulted in increased serum cytokine/chemokine levels (Fig. 5F), suggesting that the underlying GVHD was exacerbated by tumor burden. In summary, allogeneic GVT can be achieved in FL-NOG, however it comes at the expense of developing liver GVHD.

## Discussion

We present here a detailed comparison of CB-CD34^+^ and FL-CD34^+^-engrafted NOG mice, including characterization of CD34^+^ isolates, reconstitution kinetics, endpoint analyses of tissue immune cells, histopathological liver assessment and the ability to reject HLA-mismatched tumors. There are few reports that have compared CB- to FL-CD34^+^-reconstituted NSG mice with contradictory results. When equal numbers of CD34^+^ were injected, Patton et al. showed superior engraftment of FL-CD34^+^ vs. CB-CD34^+^ [14], while the opposite was observed by Chiorazzi et al. [51]. By using increasing numbers of FL-CD34^+^, another study reported a non-linear association with blood reconstitution, showing slow engraftment with < 4 × 10^4^ cells and rapid engraftment when 5 × 10^5^ cells were used [11]. We similarly show that injecting 5 × 10^4^ FL-CD34^+^ resulted in slow reconstitution kinetics (Fig. 2C) which improved substantially when we increased the cell dose to 7.5 × 10^4^ (Additional File 1: Fig. S9A).

A major confounding factor that hinders the comparisons of CB- and FL-engrafted mice is the CD34^+^ isolation method. While the isolation from CB is straightforward, the FL requires a digestion step that varies from laboratory to laboratory in terms of collagenase, time and temperature used [11, 13, 14, 40, 52–55]. Additionally, some researchers use density gradient to enrich HSPCs [13, 14, 51, 54, 55], while others do not [11, 12, 52, 53]. Some isolation techniques can enrich CD34^lo^ hepatic/mesodermal progenitors [10, 56] and whole liver extracts are known to contain non-hematopoietic CD34^+^ endothelial cells [40, 57]. Using the isolation methods described here, we show that CD34^+^ cells isolated from CB represent a relatively uniform population, while FL-CD34^+^ are composed of several distinct populations (Fig. 1), in accordance with a previous report by Chen et al. [56]. While we could identify a clear CD34^hi^CD133^hi^ population within our FL-CD34^+^ samples, the CD133^neg − lo^ could not be clearly separated into CD34^lo^CD133^lo^ and CD34^hi^CD133^neg^ as described [10, 56] (Additional File 1: Fig. S10A). Instead, these cells appeared as one CD34^+^CD133^lo − neg^ population, containing the majority of Lin^+^ (CD19^+^) and CD14^+^ cells. The CD14^+^ identified herein might be contained within the CD34^lo^CD133^lo^ described by Chen et al., given their ability to support the expansion of the CD133^hi^ FL-CD34^+^ and their potential to differentiate into endothelial cells in vitro [10]. Another discrepancy with the published research is the expression of CD45 on the FL-CD34^+^CD14^+^ population. Muench’s group has performed extensive characterization of whole fetal liver cell suspensions and has characterized the fetal liver CD14^+^ LSEC as CD45^−^ [39, 40, 58]. However, upon closer inspection, the cut-off of CD45 expression is not clearly defined in their published flow cytometry plots and could be classified as CD45^lo − int^ [40]. Our data supports a CD45^lo^ expression on the FL-CD34^+^CD14^+^ cells, which is significantly lower compared to FL-CD34^+^lin^−^ and FL-CD34^+^lin^+^ counterparts (Fig. 1D and Additional File 1: Fig. S6A).

Two recent studies have explored the transcriptome of fetal liver cells on a single-cell level [41, 59]. The study by Popescu et al. identified three main populations expressing CD34, that could be potentially isolated using anti-CD34 magnetic beads: (i) hematopoietic stem cells and multipotent progenitors (MSC/MPP), (ii) CD19^+^ Pre B cells and (iii) CD14^+^ endothelial cells [41]. To verify that these correspond to the (i) lin^−^ HSPC, (ii) lin^+^CD38^high^ and (iii) CD14^+^ endothelial populations identified in our FL-CD34^+^ isolates, we performed additional staining of CD19 and endothelial markers CD31 and CD144. We confirmed that the major lineage markers expressed by FL-CD34^+^ are CD19 and CD14 (Additional File 1: Fig. S6B) and that CD14^+^ cells co-express CD31 and CD144 (Fig. 1F and Additional File 1: Fig. S6C, D). VE-cadherin (CD144) was specific for CD14^+^ cells, while CD31 expression was significantly higher on CD34^+^lin^−^ cells (Fig. 1F and Additional File 1: Fig. S6C-D). This is in accordance with a previous study showing CD31 expression on hematopoietic stem cells throughout ontogeny, including the fetal liver stage [60]. Beside CD14, Popescu et al. identified the expression of CD4 on fetal endothelial cells [41]. Upon verifying and confirming an exclusive CD4 expression (Fig. 1E, F and Additional File 1: Fig. S6D), these cells could be specifically characterized as FL-CD34^+^CD14^+^CD144^+^CD4^+^.

By comprehensive phenotyping of FL-CD34^+^ cells, Vanuytsel et al. identified a CD19^+^ B lineage precursor cluster characterized by CD38^high^ expression [61]. These cells are likely equivalent to the lin^+^ (CD19^+^) population described here (Fig. 1A-E) and the CD19^+^CD34^+^ Pre B cells from the study by Popescu et al. [41]. Lineage committed cells progressively lose their proliferative capacity [62]. A significant proportion of lineage committed CD19^+^ cells within FL-CD34^+^ might explain a slower reconstitution of the human immune system in FL-NOG compared to CB-NOG, especially B cells (Figs. 1A and B and 2D and E). The rest of FL-CD34^+^ from Vanuytsel et al. were composed of several HSPC clusters [61], equivalent to the FL-CD34^+^lin^−^ described here. Within these lin^−^ HSPC we detected higher myeloid progenitor frequencies in FL-CD34^+^ compared to CB-CD34^+^, which upon transplantation resulted in an increased proportion of CD33^+^ myeloid cells in the blood of FL-NOG. Additionally, FL-NOG mice consistently harboured higher numbers of CD16^hi^CD66b^hi^ neutrophils across organs. This is likely due to increased development in the bone marrow and warrants further investigation in models that support better neutrophil reconstitution, such as hG-CSF KI NOG [63]. FL-NOG spleens, lungs and liver also contained more macrophages and dendritic cells, possibly due to higher myeloid progenitor frequencies. However, the increased myeloid cell infiltration is more likely associated with the inflammatory environment that we uncovered in FL-NOG because of CD14^+^ endothelial cell transplantation (Fig. 4).

CD34^+^CD14^+^ endothelial cells represent approximately 2–4% of total digested fetal liver cells [41], however their enrichment within CD34^+^ isolates appears to vary considerably among studies. Despite the similarity of FL-CD34^+^ isolation protocols, our samples consistently contained between 2 and 20% CD14^+^ cells (Fig. 1B), while Vanuytsel et al. reported only a minor endothelial cell component [59]. Possible reasons for the observed discrepancy in the frequency of CD14^+^ cells compared to Vanuytsel et al. [59], include a different type of density gradient media and the use of a different CD34 isolation kit. In our experience with Ficoll-paque PLUS, the mononuclear layer upon density centrifugation could not be completely separated from the layer below. This might have contributed to the presence of CD34^+^CD14^+^ cells within the FL-CD34^+^ isolates. A recent study by Kaffe et al. suggests that such endothelial contamination might be a common occurrence. In their study, the authors isolated FL-CD34^+^ in a similar fashion and upon engraftment in MISTRG6 mice detected FVIII-producing LSECs and other non-parenchymal cells (NPCs) within the liver [45]. It remains to be determined if the precursors of NPCs contain the FL-CD34^+^CD14^+^ described here. CD14^+^ LSECs from fetal liver have been shown to engraft in urokinase-type plasminogen activator transgene (uPA-NOG) mice and produce FVIII [39]. Here we show for the first time that FL-CD34^+^CD14^+^ LSECs can engraft in simple NOG mice and enhance the reconstitution of the human immune cells in this strain. Removing CD14^+^ cells from FL-CD34^+^ before transplantation significantly delayed the engraftment of the human immune system in NOG mice, specifically affecting CD19^+^ and CD33^+^ cell numbers (Fig. 2E, I). LSECs have been shown to promote HSPC expansion ex vivo [44] and may play a supportive role in hematopoietic stem cell engraftment and differentiation in vivo through the secretion of factors like Stromal-derived factor (SDF)-1/CXCL12 [64]. The FL-CD34^lo^CD133^lo^ identified by Chen et al. that might contain the FL-CD34^+^CD14^+^ (Additional File 1: Fig. S10) have also been shown to express growth factors CXCL12, DLK1 and CSF that support the expansion of human HSC in vitro and in vivo [10]. Another possibility is that human LSEC engraftment induces some sort of damage to the mouse sinusoidal endothelium. This could lead to immune activation and recruitment of immune cells into the liver and other organs through the secretion of mediators such as CCL2 and CXCL10, ultimately resulting in liver injury [49].

**Fig. 6:**
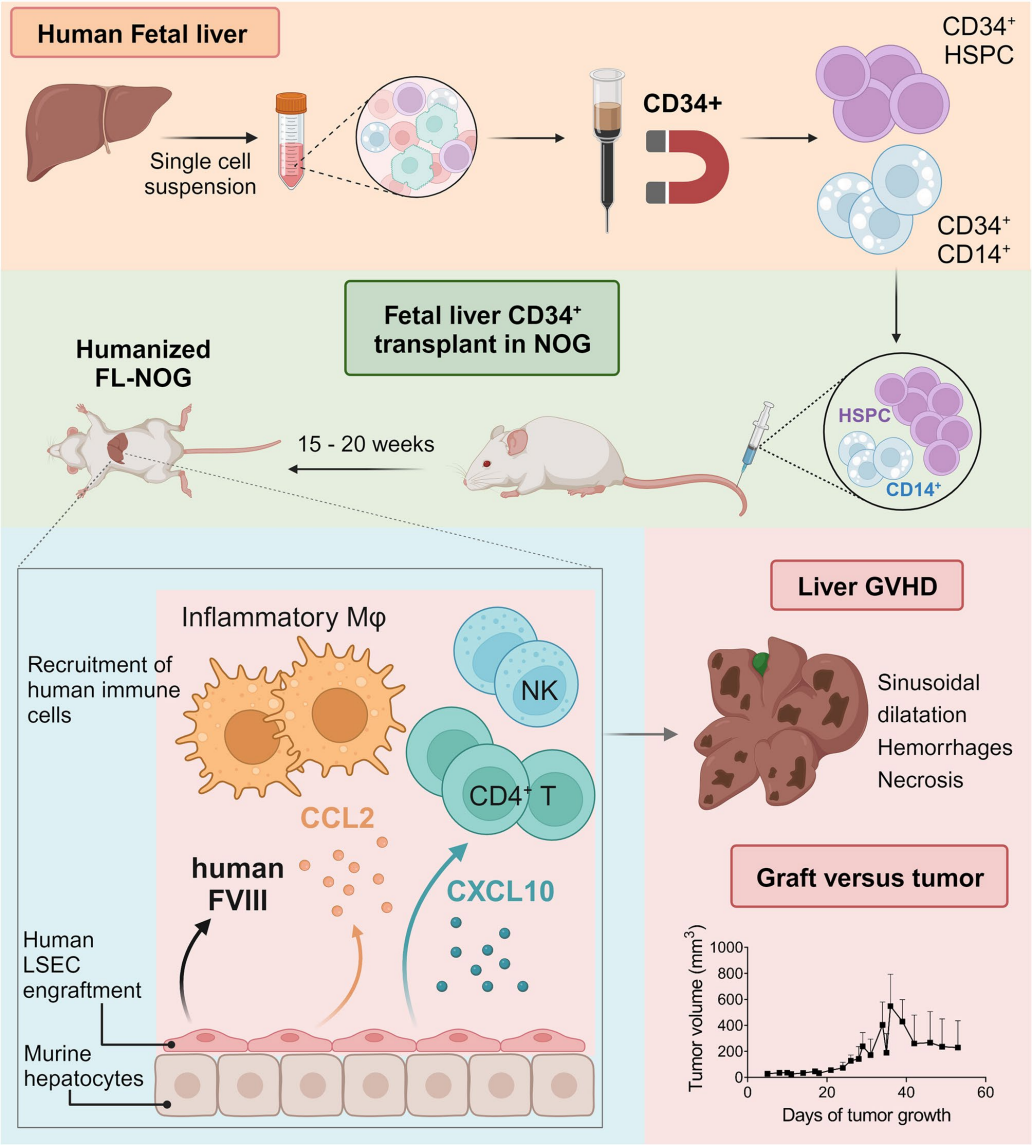
Proposed mechanism of spotted liver GVHD development with associated GVT in humanized NOG mice reconstituted with FL-CD34^+^. Created with BioRender.com

Long-term reconstitution of immune cells can cause spontaneous activation of the human immune system in humanized mice, resulting in different manifestations of xenogeneic GVHD. CD34^+^ isolated from CB and GM-CSF-mobilized peripheral blood have been shown to induce a form of skin GVHD (GVH-S) characterized by alopecia and scaly skin with or without weight loss [24, 25, 48, 65]. GVH-S develops in a donor-specific manner and seems to be associated with certain HLA haplotypes [48, 65]. Our study confirmed donor-dependent facial alopecia in CB-NOG associated with increased T and NK cell numbers in the spleen and liver. Another type of GVHD with multisystemic granulomatous has been identified in mice reconstituted with G-CSF-mobilized peripheral blood (MBP)-CD34^+^ [66, 67], CB-CD34^+^ [68] and FL-CD34^+^ [12]. T cells and macrophages have been found in BM, liver and lung infiltrates of these animals [12, 67, 68]. The liver pathology observed in FL-NOG from our study was similarly correlated with both CD4^+^ T cells and macrophages, specifically CD14^high^CD68^+^ that correspond to the inflammatory CD32^mid^ Kupffer cells identified by Wu et al. [69]. The increased soluble factors, including CXCL10 and CCL2, likely contributed to the recruitment of T and NK cells and egress of myeloid cells from the BM resulting in increased organ infiltration (Fig. 6). We have uncovered that sinusoidal dilatation (SD) was the most distinctive feature present in FL-NOG livers. SD can be caused by endothelial dysfunction, which is a common complication associated with tissue rejection [70–72]. Taken together, these findings suggest that the spotted liver phenotype is likely a form of GVHD [73, 74]. Removing endothelial CD14^+^ cells from the FL-CD34^+^ preparations not only delayed blood reconstitution, but also significantly reduced serum CXCL10 and CCL2 and human cell infiltration into the livers of transplanted NOG mice. Consequently, none of the FL-CD14^−^-NOG developed the spotted liver phenotype. In the case of infection or injury, LSEC regulate the recruitment of leukocytes into the liver tissue by producing chemoattractants like CXCL10 and CCL2 [49, 75]. However, we cannot exclude that these chemokines are produced by the human immune cells themselves and the observed liver pathology warrants further exploration that is beyond the scope of the current study.

The involvement of FL-CD34^+^CD14^+^ engraftment in the development of GVHD in humanized mice has not been previously reported as not many studies knowingly transplanted CD14^+^ fetal cells into immunocompromised mice. To the authors knowledge, the studies from Muench’s group are the only reports focused on fetal CD14^+^ LSEC characterization, transplantation, and engraftment into immunodeficient mice [39, 40, 58]. In these reports, the authors did not examine pathological changes caused by CD14^+^ engraftment. Additionally, one of these studies specifically excluded animals exhibiting GVHD signs such as hair loss and/or splenomegaly associated with high CD3^+^ T cell numbers [58]. The development of GVHD might be linked to the route of administration that can impact cell homing in various niches and might cause pulmonary sequestration [76, 77]. Tail vein or retro-orbital injections of FL-CD34^+^ together with the implantation of autologous fetal liver (FL) and fetal thymus fragments under the renal capsule are used to generate “bone marrow, liver, thymus” (BLT) humanized models [15, 78, 79]. BLT mice often develop a wasting chronic GVHD, which can appear as early as 17–20 weeks post engraftment and can significantly limit the experimental window for their use [15, 78]. On the contrary, groups that routinely use intra-hepatic injection of FL-CD34^+^ into newborn mice, do not report the development of xenogeneic GVHD [11, 13, 45, 56]. This suggests that intra-hepatic transplantation might be more physiological since the human fetal progenitors home into a neonatal (albeit mouse) environment [76]. However, when a study aimed specifically at identifying pathological features upon intra-hepatic neonatal FL-CD34^+^ transplantation, it unravelled that 20% of mice developed multisystemic granulomatous inflammatory infiltrates [12]. As in our study, these infiltrates consisted predominately of human macrophages and T cells and led to 7% mortality and morbidity within 3–6 months [12]. By transplanting adult mice via the tail vein, we similarly recorded one death out of 14 mice (7%) (Additional File 2: Table S3).

Another important factor to consider is the study duration since the liver lesions described here were not visible until 20 weeks post-engraftment. Importantly, except for one mouse (Additional File 2: Table S3), none of the mice died and did not suffer from weight loss or other visible signs of GVHD. Mice became moribund only when an additional pro-inflammatory factor was added (tumor burden) after long-term reconstitution (≥ 20 days post-transplantation) (Additional File 1: Fig.S9C-E). Thus, it is not surprising that studies with shorter endpoints such as 12 weeks post-engraftment do not report a similar phenotype, despite showing evidence of FL-CD34^+^-derived LSEC engraftment [45].

Finally, we investigated the functionality of the human immune system in CB- and FL-NOG by engrafting an HLA-A-mismatched melanoma CDX. We observed complete tumor rejection in ≥ 50% of FL-NOG or FL-NOG-A24 Tg, but not in CB-NOG. The HLA-A24 Tg was not necessary for the tumor rejection and majority of the mice developed the spotted liver GVHD phenotype. These observations suggests that the graft-versus-tumor (GVT) effect is either due to a better functionality of the immune system, associated with the GVHD responses or a combination of both [80]. The goal of allogeneic hematopoietic cell transplantation in a clinical setting is to achieve GVT without inducing significant GVHD [80]. In our study, we observed a severe wasting GVHD leading to experimental animal loss, primarily in mice that failed to reject tumors. This suggests that the underlying GVHD can be intensified by tumor burden or possibly by hypercytokinemia induced by the ongoing tumor lysis [81]. Due to the overlapping characteristics of these conditions, further studies are needed to elucidate the mechanisms involved in endothelial dysfunction, GVHD and GVT in FL-NOG mice.

Among the limitations of the present study is the lack of a FL-CD34^+^CD14^+^ group to determine if these cells can engraft in the mouse liver without the presence of hematopoietic cells. Additionally, it would be beneficial to include an HLA-A matched tumor group to test whether the observed tumor rejection is at least in part due to an allogeneic reaction or is a consequence of the GVHD reaction. This will be addressed in future research alongside the development of an alternative protocol for the isolation of FL-CD14^−^ HSPCs. One approach would be to use CD133 positive selection to enrich for the CD133^hi^CD34^hi^ population. Increasing the cell dose for transplantation might overcome the need for CD14^+^ cells for successful engraftment and avoid the development of liver disease. Finally, if neonatal mice become available to us, we could get valuable insights into homing, engraftment and GVHD development by performing a comparative study of neonatal intra-hepatic and adult intravenous administration of FL-CD34^+^ cells from the same donors.

## Conclusions

Taken together our study highlights the importance of deeper phenotyping of FL-CD34^+^ isolates prior transplantation. As we have shown here, the FL contains multiple CD34^+^-expressing populations that can have a profound impact on the phenotypes of transplanted animals. Our study provides insights into a novel form of GVHD that develops in FL-NOG, which can be attributed to the CD14^+^ endothelial cells contained within FL-CD34^+^ isolates (Fig. 6). Finding strategies to prevent or delay the loss of immunological tolerance towards the host is crucial for the development of models requiring stable long-term engraftment. On the other hand, FL-NOG might serve as a unique model to study GVT responses and possible strategies to prevent the underlying endothelial dysfunction and GVHD.

### Electronic supplementary material

Below is the link to the electronic supplementary material.

Supplementary Material 1

Supplementary Material 2

## Data Availability

The datasets used and/or analysed during the current study are available from the corresponding author on reasonable request.

## Declarations

All cord blood and fetal liver donations were performed in compliance with the SingHealth Centralised Institutional Review Board (CIRB) guidelines and written informed consent for tissue donation was obtained from all donors. CIRB #2013/778/D titled “Derivation of Stem Cells from Perinatal Birth Tissues” CIRB #2013/837/D titled “Ontogeny of the Fetal Immune System” were first approved in November 2013 and both protocols are renewed yearly. For human plasma sample collection, the study was approved by the National Healthcare Group (NHG) Domain Specific Review Board (#DSRB-2011/01917). Written informed consent was obtained from the participants before recruitment.

## Abbreviations

ALT: Alanine transaminase
BM: Bone marrow
CB: Cord blood
CB-NOG: NOG mice transplanted with CB-CD34^+^
CDP: Common dendritic cell progenitors
CDX: Cell line-derived xenograft
DC: Dendritic cell
FL: Fetal liver
FL-NOG: NOG mice transplanted with FL-CD34^+^
FL-CD14^−^- NOG: NOG mice transplanted with CD14^+^-depleted FL-CD34^+^
FMO: Fluorescence minus one
FVIII: Factor VIII
GVHD: Graft-versus-host disease
GVH-S: Skin graft-versus-host
GVT: Graft-versus-tumor
H&E: Haematoxylin and Eosin
HIS: Humanized immune system
HLA: Human leukocyte antigen
HSPC: Hematopoietic stem and progenitor cells
Lin: Lineage
LSEC: Liver sinusoidal endothelial cells
MDP: Monocyte-DC progenitors
NOG: NOD/Shi*-scid Il2rg*^*null*^ mice
NSG: NOD/LtSz-*scid Il2rg*^*null*^ mice
PDX: Patient-derived xenograft
PBS: Phosphate buffered saline
SD: Sinusoidal dilatation
Tg: Transgenic
uPA-NOG: NOG mice expressing transgenic urokinase-type plasminogen activator in the liver
wpt: Weeks post-transplant
