# Supplementary material for: Fetal liver CD34+ contain human immune and endothelial progenitors and mediate solid tumor rejection in NOG mice
Source: Stem Cell Res Ther. 2024 Jun 9;15:164. doi: 10.1186/s13287-024-03756-7 (PMC11163708; doi:10.1186/s13287-024-03756-7)
Supplement: Supplementary file 1 — Supplementary Material 1 [file 13287_2024_3756_MOESM1_ESM.pdf]

Additional File 1

Supplemental Figures

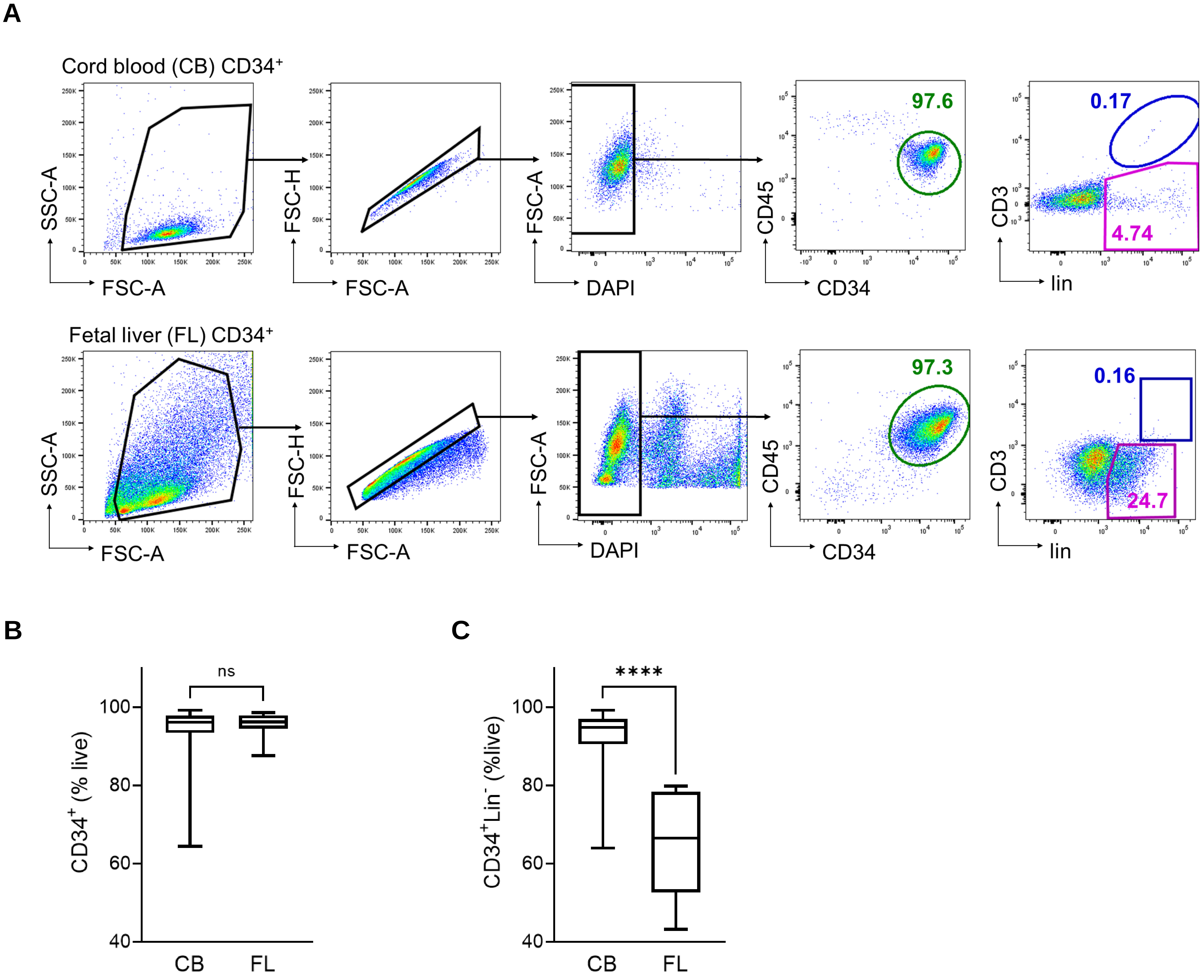

Figure S1. (A) Gating strategy for assessing the purity of CD34⁺ freshly isolated from cord blood (upper panel) and fetal liver (lower panel). (B) Purity measured as percentage of CD34⁺ within the live cells in cord blood (CB) and fetal liver (FL). (C) Purity measured as percentage CD34⁺ lineage (CD3, CD14, CD16, CD19, CD20, CD56) negative (lin⁻) within the live cells in CB and FL. Box plots showing minimum, maximum and median value of n = 64 (cord blood) and n = 7 (fetal liver).

Gated on Singlet, live:

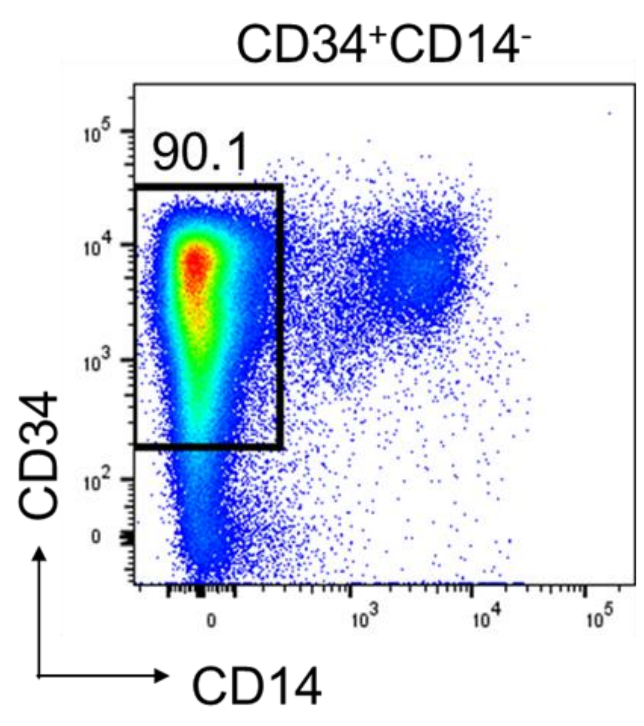

Figure S2. Gating strategy for the sorting of FL-CD34^{+}CD14^{-}. The number of FL-CD34^{+}CD14^{-} cells to inject was calculated by multiplying the percentage of CD34^{+}CD14^{-} with the total FL-CD34^{+} injected/mouse; in this case 90% x 50.000 = 45.000/mouse.

Gated on Singlet, live:

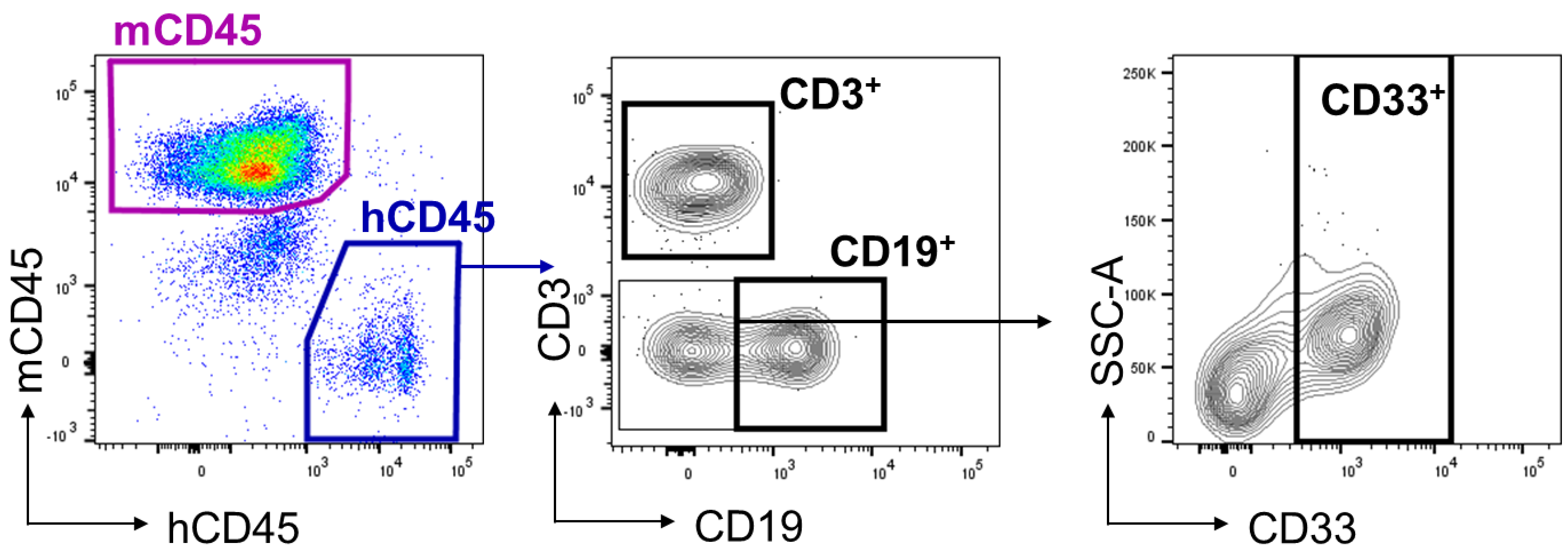

Figure S3. Gating strategy for the assessment of the reconstitution of human CD45 (hCD45), CD3+T cells, CD19+ B cells and CD33+ myeloid cells in the blood of humanized mice.

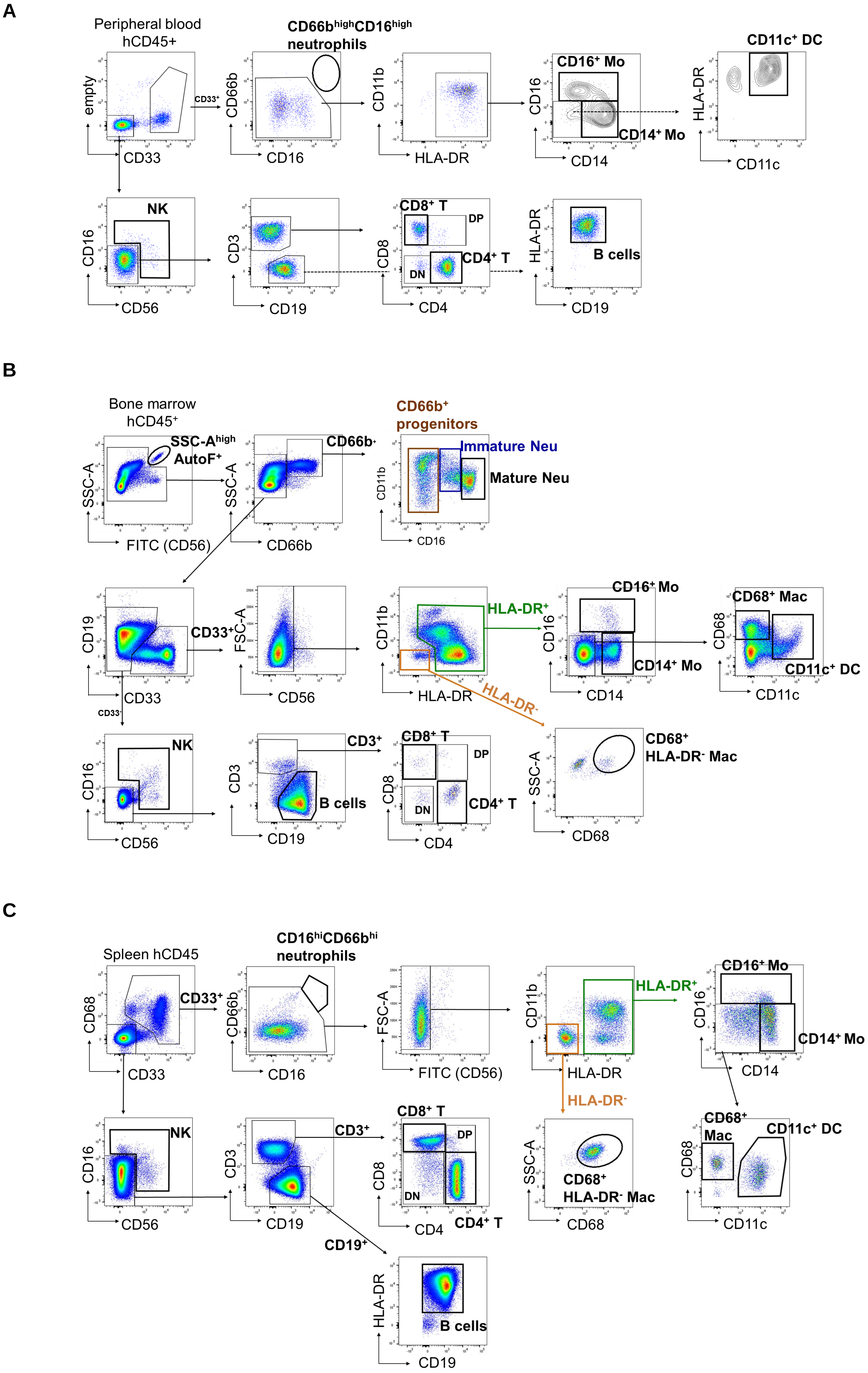
A
Peripheral blood hCD45+
empty
CD33
CD33+
CD66bhighCD16high neutrophils
CD66b
CD16
CD11b
HLA-DR
CD16+ Mo
CD14+ Mo
CD14
CD11c+ DC
CD11c
NK
CD56
CD3
CD19
CD8+ T
DP
DN
CD4+ T
CD8
CD4
B cells
B
Bone marrow hCD45+
SSC-Ahigh AutoF+
SSC-A
FITC (CD56)
CD66b+
CD66b
CD66b+ progenitors
Immature Neu
Mature Neu
CD11b
CD16
CD19
CD33+
CD33
CD33-
FSC-A
CD56
HLA-DR+
HLA-DR-
HLA-DR
CD16+ Mo
CD14+ Mo
CD14
CD68+ Mac
CD68
CD11c+ DC
CD11c
NK
CD3+
CD3
B cells
CD8+ T
DP
DN
CD4+ T
CD8
CD4
SSC-A
CD68+ HLA-DR- Mac
C
Spleen hCD45
CD68
CD33
CD33+
CD16hiCD66bhi neutrophils
CD66b
CD16
FSC-A
FITC (CD56)
CD11b
HLA-DR
HLA-DR+
HLA-DR-
CD16+ Mo
CD14+ Mo
CD14
NK
CD56
CD3+
CD3
CD19
CD19+
CD8+ T
DP
DN
CD4+ T
CD8
CD4
SSC-A
CD68+ HLA-DR- Mac
CD68+ Mac
CD11c+ DC
CD11c
HLA-DR
B cells

D

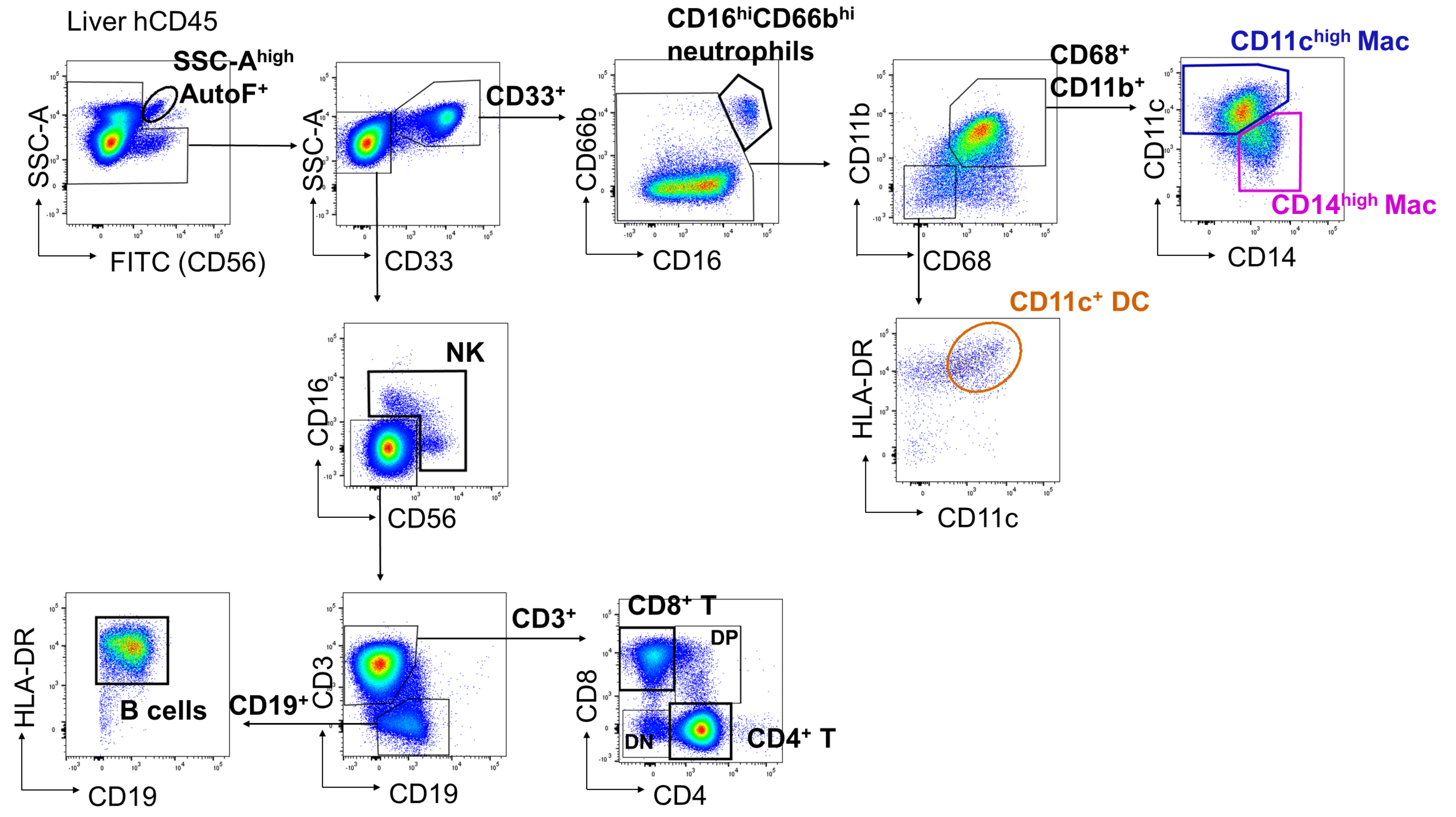

E

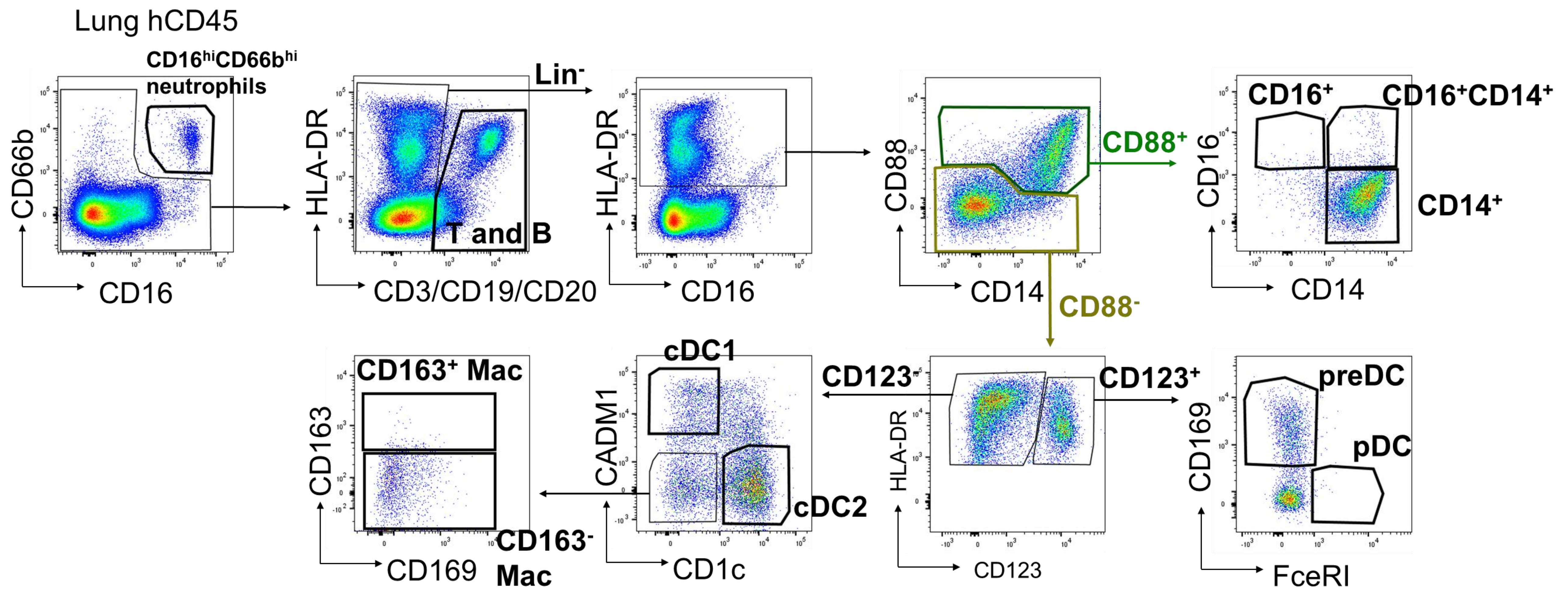

Figure S4. Gating strategies for the analysis of human CD45 subsets in (A) peripheral blood, (B) bone marrow, (C) spleen, (D) liver and (E) lung.

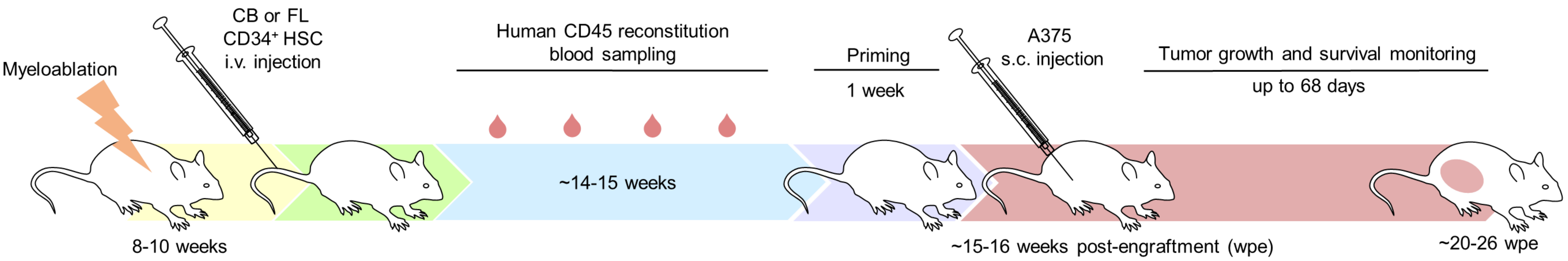

Figure S5. Schematic representation of the A375 melanoma CDx rejection model.

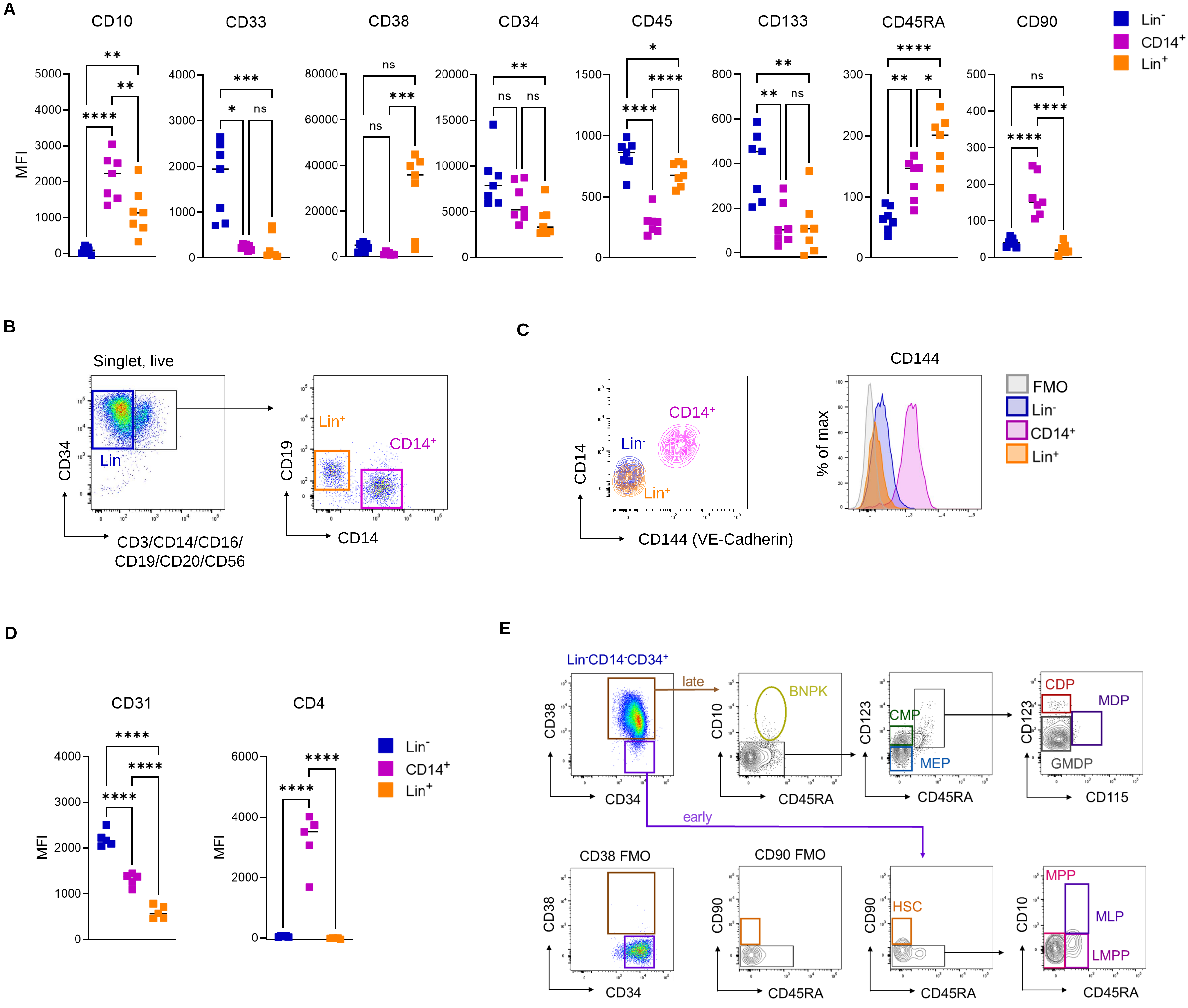

Figure S6. (A) Quantification of the expression of the indicated markers on the surface of the three populations (Lin-, CD14+ and Lin+) identified within FL-CD34+ (n = 7; MFI: median fluorescence intensity). (B) Gating strategy to assess the expression of (C) VE-Cadherin (CD144) on Lin-, CD14+ and Lin+ FL-CD34+. (D) Expression of CD31 and CD4 on Lin-, CD14+ and Lin+ FL-CD34+ quantified as MFI (n = 5). Each symbol represents one donor; groups were compared with ordinary one-way ANOVA or Kruskal-Wallis test with post-hoc Tukey or Dunn's, respectively. *p < 0.05, **p < 0.01, ***p < 0.001, ****p < 0.0001. (E) Gating strategy used to analyze the progenitor populations contained within Lin- CB- or FL-CD34+. Abbreviations: FMO: fluorescence minus one; HSC: hematopoietic stem cells; MPP: multipotent progenitor; LMPP: lymphoid-primed multi-potent progenitor; LMPP: lymphoid-primed multi-potent progenitor; MLP: multi-lymphoid progenitor; BNPK: B/NK progenitor; CMP: common DC progenitor; GMDP: granulocyte-monocyte-DC progenitor; MDP: monocyte-DC progenitor; CDP: common DC progenitor; MEP: megakaryocytic-erythroid progenitors.

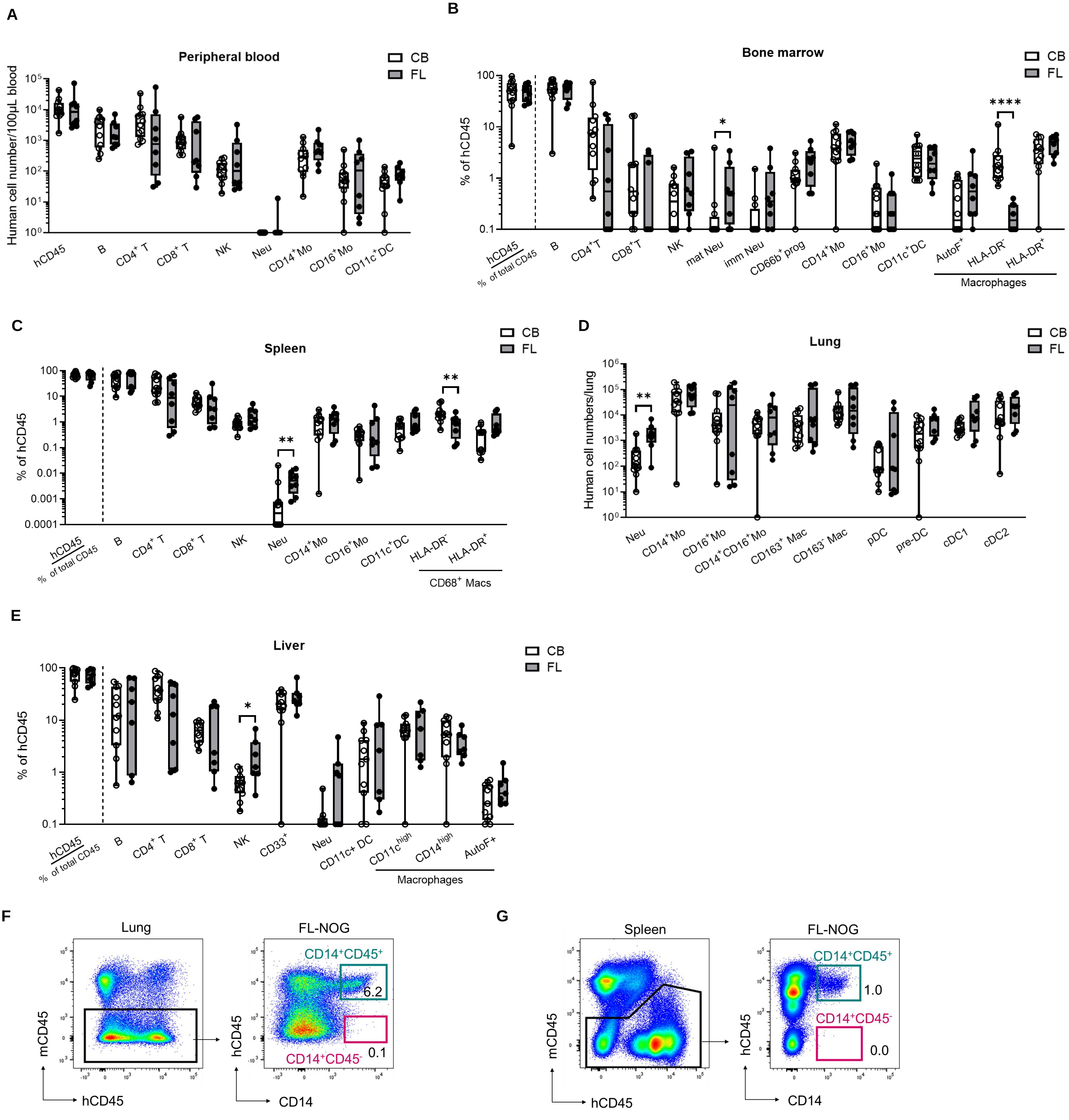

Figure S7. Mice were euthanized 20 weeks after transplantation and peripheral blood, bone marrow (1 femur), spleen, lung and liver were collected and analysed by flow cytometry. (A) Numbers of human immune cells in peripheral blood and (B) frequencies in bone marrow. (C) Percentages of human immune cells in spleen, (D) numbers of human immune cells in the lung and (E) frequencies in the liver. Flow cytometry plots of (F) lung and (G) liver from a representative FL-NOG mouse showing no engraftment of $CD14^{+}CD45^{-}$ endothelial cells. Box and whiskers plots showing all points from min to max, each symbol represents a single mouse (n = 12 for CB and n = 8 for FL). Statistical comparisons by Mann-Whitney tests, *p < 0.05, **p < 0.01, ***p < 0.001, ****p < 0.0001.

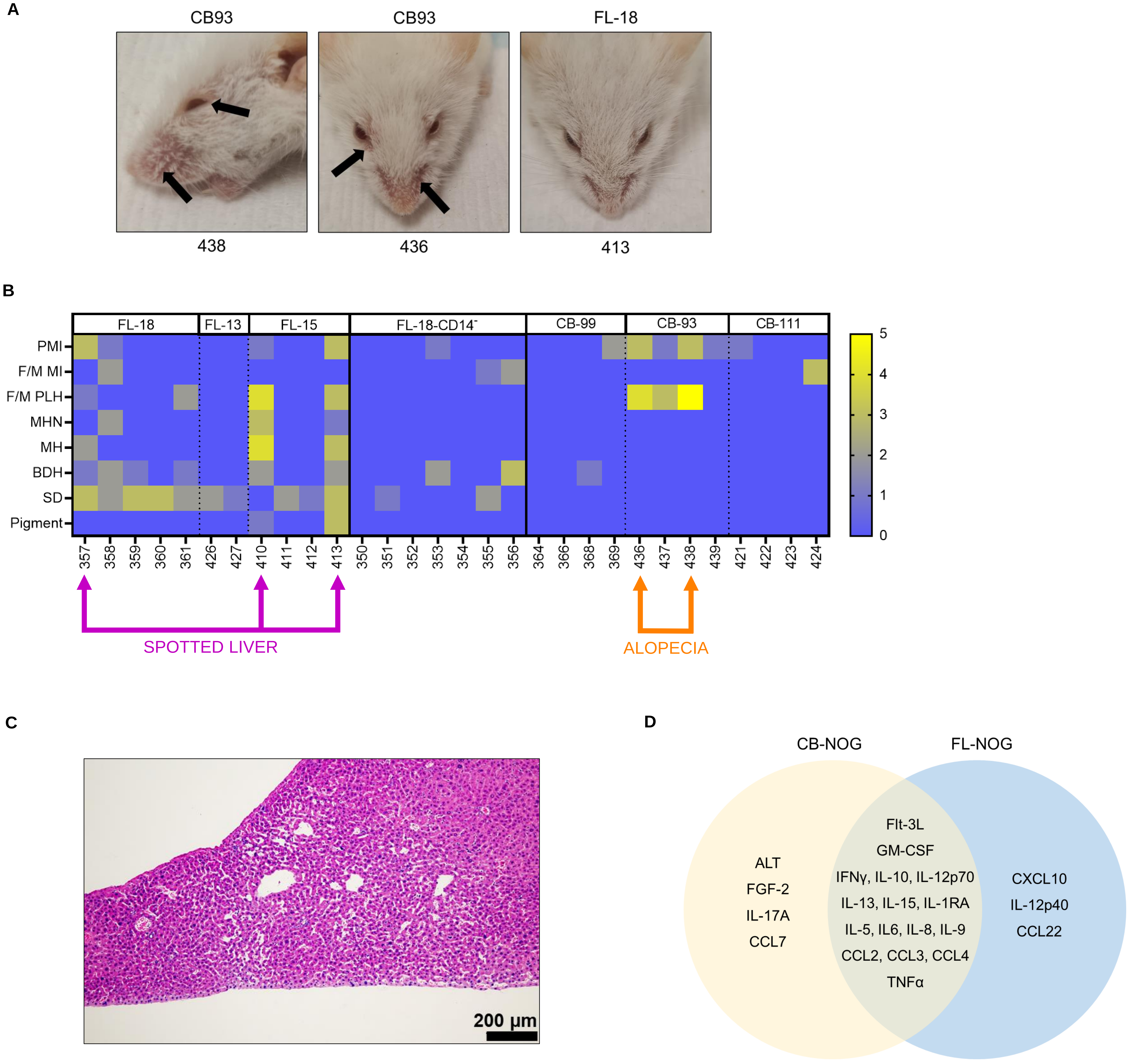

Figure S8. (A) Photos of mice showing facial alopecia with donor code above (CB93 and FL-18) and individual mouse ID indicated below. (B) Heatmap showing the pathological score (0-5) for the following pathological features: portal mononuclear (granulomatous, lymphoplasmacytic) inflammation (PMI), Focal/Multifocal mononuclear inflammation (F/M MI), Focal/Multifocal pyogranulomatous, lymphoplasmacytic hepatitis with/without giant cells (F/M PLH), Multifocal hepatic necrosis (MHN), Multifocal hemorrhages (MH), Bile duct hyperplasia (BDH), sinusoidal dilatation (SD). Donor IDs on top and individual mice IDs indicated below. Purple arrows pointing to the mice that developed spotted liver and orange arrows pointing to mice that developed facial alopecia. (C) Representative liver H&E section with sinusoidal dilatation (SD) without any associated pathologies. (D) Venn diagram summarizing mediators correlating with liver pathology exclusively in CB-NOG, FL-NOG or both.

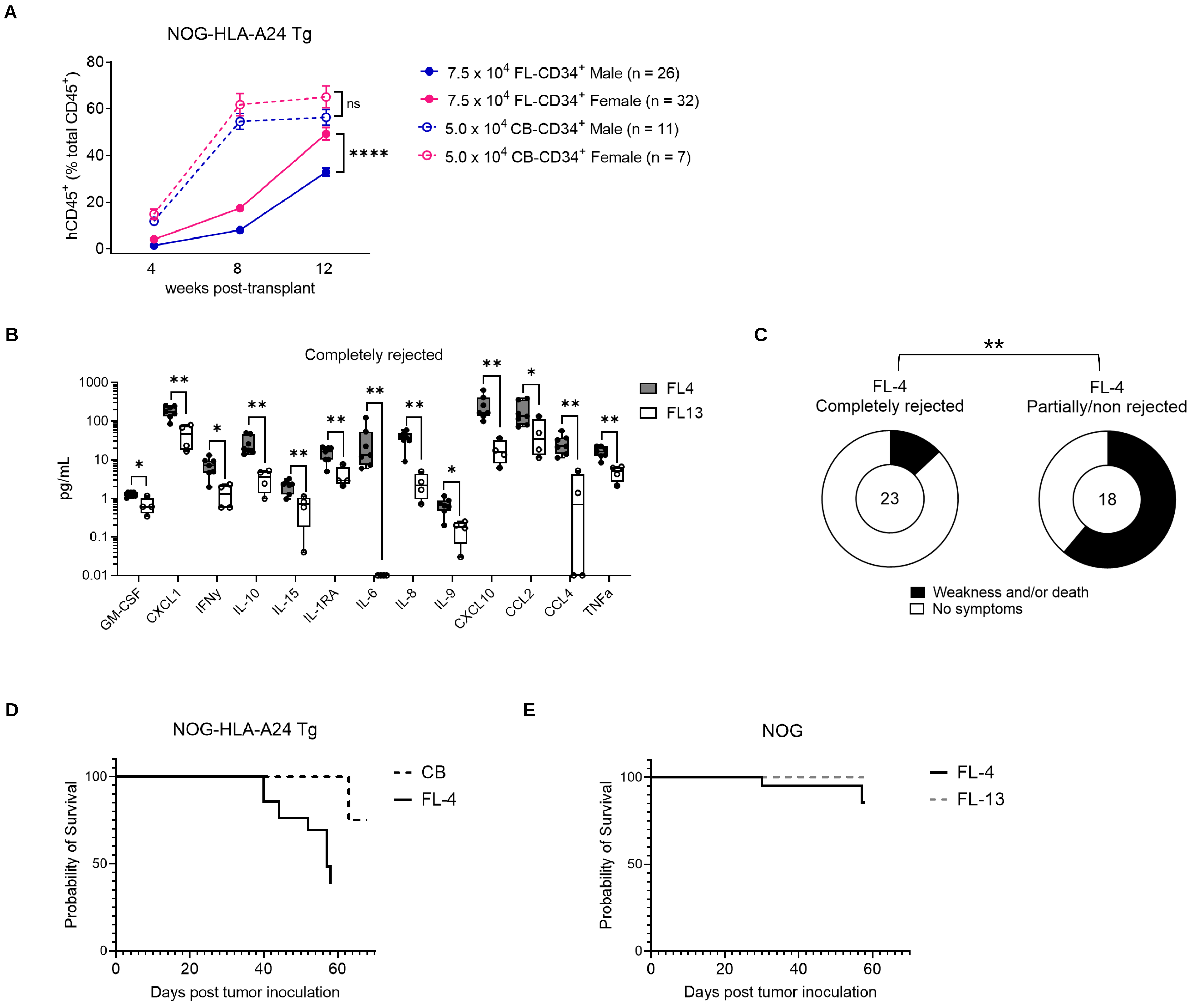

Figure S9. (A) Reconstitution kinetics in the peripheral blood of male (blue) or female (magenta) NOG-HLA-A24 Tg mice transplanted with either 5 x 10^4 CB-CD34$^+$ or 7.5 x 10^4 FL-CD34$^+$. (B) Serum cytokines and chemokines in NOG mice reconstituted with either donor FL4 or FL13, that completely rejected the A375 CDx. Box and whiskers plots showing all points from min to max, each point representing one mouse (n = 7 for FL-4 and n = 4 for FL-13). Statistical comparisons among two groups by Mann-Whitney tests, *$p < 0.05$, **$p < 0.01$. (C) Donut-charts showing the proportion of mice that developed weakness and had to be euthanized on humane grounds or were found dead in FL4-reconstituted NOG with (partially/non-rejected) or without (completely rejected) tumor burden. Total n for each condition indicated in the center of the donut charts. Significance was computed with the Fischer's exact test, **$p < 0.01$. (D) Survival curves of NOG-HLA-A24Tg mice reconstituted with CB-CD34$^+$ (CB, n = 7) or FL-CD34$^+$ (FL-4; n = 21). (E) Survival curves of NOG-HLA-A24Tg reconstituted with FL-4 (n = 20) or FL-13 (n = 13) FL-CD34$^+$.

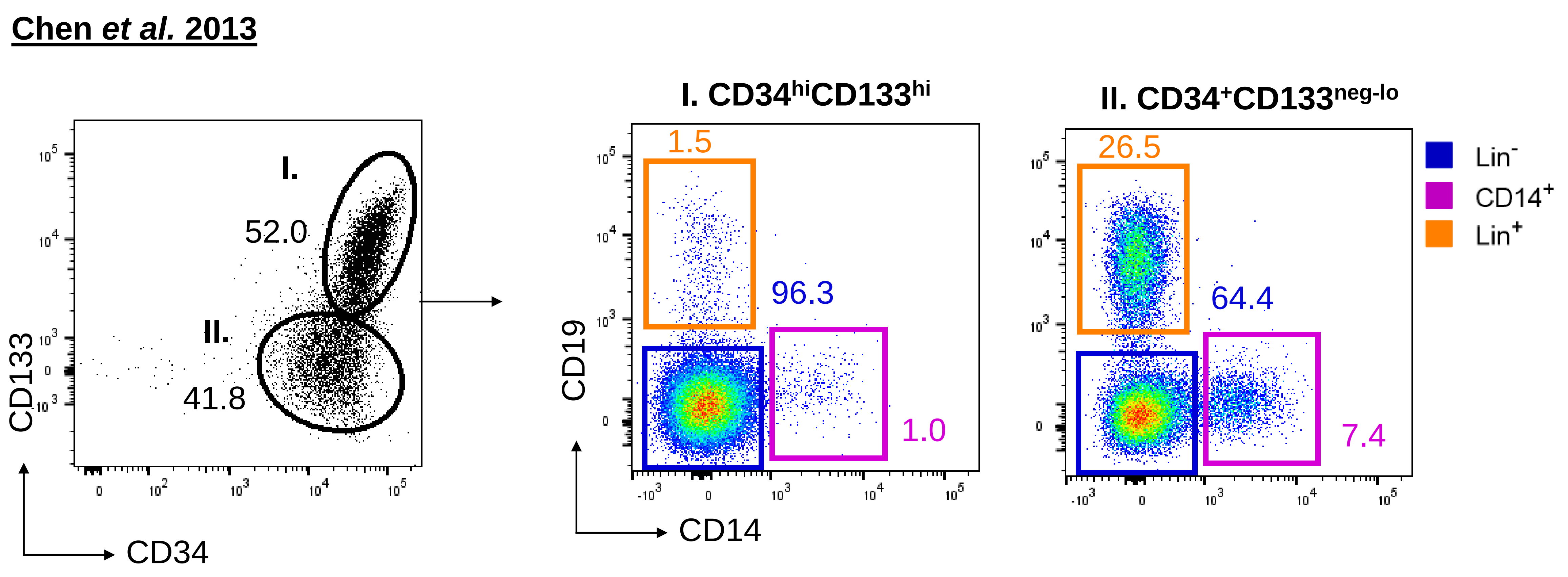

Figure S10. Gating of FL-CD34+ cells described in this study according to the strategy defined by Chen *et al*, 2013. Flow cytometry plots were generated in FlowJo by concatenating equal number of events from 5 separate FL-CD34+ samples, pre-gated on singlet, live cells. Cells were stained with the panel in Additional File 2: Table S2C as described in the Method section under “Flow Cytometric analysis of CB- and FL-CD34+”.
