# Supplementary material for: Fetal liver CD34+ contain human immune and endothelial progenitors and mediate solid tumor rejection in NOG mice
Source: Stem Cell Res Ther. 2024 Jun 9;15:164. doi: 10.1186/s13287-024-03756-7 (PMC11163708; doi:10.1186/s13287-024-03756-7)
Supplement: Supplementary file 2 — Supplementary Material 2 [file 13287_2024_3756_MOESM2_ESM.pdf]

Additional File 2

Supplemental Tables

Table S1. Antibodies for assessing CD34^{+} HSCP purity

Antigen	Fluorochrome	Reactivity	Clone	Supplier	Catalog Number
Lineage (CD3, CD14, CD16, CD19, CD20, CD56)	FITC	anti-human	UCHT1; HCD14; 3G8; HIB19; 2H7 ; HCD56	Biolegend	348801
CD38	PE	anti-human	HIT2	Biolegend	303506
CD34	PE-Cy7	anti-human	581	Biolegend	343516
CD3	APC	anti-human	UCHT1	Biolegend	300412
CD45	APC-Cy7	anti-human	2D1	Biolegend	368516

Table S2. Antibody panels for CD34^{+} HSPC phenotyping

A. CD34^{+} HSPC phenotyping panel					
Antigen	Fluorochrome	Reactivity	Clone	Supplier	Catalog Number
CD33	BUV395	anti-human	WM53	BD	740293
CD14	BUV737	anti-human	M5E2	BD	612763
CD133	BV786	anti-human	W6B3C1	BD	747640
CD3	BV650	anti-human	SP34-2	BD	563916
CD16	BV650	anti-human	3G8	BD	563692
CD19	BV650	anti-human	SJ25C1	BD	563226
CD20	BV650	anti-human	2H7	BD	563780
CD56	BV650	anti-human	NCAM16.2	BD	564057
CD45	BV510	anti-human	HI30	Biolegend	304036
CD90	BV421	anti-human	5E10	Biolegend	328122
CD45RA	FITC	anti-human	5H9	BD	556626
CD34	PE-Cy7	anti-human	581	Biolegend	343516
CD123	PE/Dazzle594	anti-human	6H6	Biolegend	306034
CD38	PE	anti-human	HIT2	Biolegend	303506
CD10	APC-Cy7	anti-human	HI10a	Biolegend	312212
CD115	APC	anti-human	9-4D2-1E4	Biolegend	347306
B. FL-CD34^{+} VE-Cadherin panel					
Antigen	Fluorochrome	Reactivity	Clone	Supplier	Catalog Number
Lineage (CD3, CD14, CD16, CD19, CD20, CD56)	FITC	anti-human	UCHT1; HCD14; 3G8; HIB19; 2H7 ; HCD56	Biolegend	348801
CD144	PE	anti-human	BV9	Biolegend	348505
CD34	PE-Cy7	anti-human	581	Biolegend	343516
CD14	Alexa Fluor 700	anti-human	M5E2	BD	557923
CD19	BV650	anti-human	SJ25C1	BD	563226
CD45	BV510	anti-human	HI30	Biolegend	304036
C. FL-CD34^{+} CD31 and CD4 panel					
Antigen	Fluorochrome	Reactivity	Clone	Supplier	Catalog Number
CD38	FITC	anti-human	HIT2	Biolegend	303504
CD19	PE	anti-human	HIB19	Biolegend	302208
CD31	Alexa Fluor 594	anti-human	WM59	Biolegend	303126
CD34	PE-Cy7	anti-human	581	Biolegend	343516
CD4	APC-Cy7	anti-human	RPA-T4	BD	557871
CD14	Alexa Fluor 700	anti-human	M5E2	BD	557923
CD133	BV786	anti-human	W6B3C1	BD	747640
CD20	BV650	anti-human	2H7	BD	563780
CD45	BV510	anti-human	HI30	Biolegend	304036

Table S3. Details of experimental mice used for the comparison of CB-CD34^{+} and FL-CD34^{+} engraftment

Cohort/ Independent experiment	mouse ID	donor ID	Experimental animal death	endpoint (weeks post-transplant)	Analyzed for Fig. 2B-I	Analyzed for Fig 2J	Analyzed for Fig 3A-F (20 weeks) and Fig S7	Analyzed for Fig 3G-I	Analyzed for Pathology Fig 4B,D	Analyzed for correlation with pathology_20wks only Fig. 4E-H	Analyzed for Fig 4J
1	NOG__350	FL-18 CD14-		15	yes	yes		yes	yes		yes
	NOG__351	FL-18 CD14-		15	yes	yes		yes	yes		yes
	NOG__352	FL-18 CD14-		20	yes	yes		yes	yes		yes
	NOG__353	FL-18 CD14-		15	yes	yes		yes	yes		yes
	NOG__354	FL-18 CD14-		20	yes	yes		yes	yes		yes
	NOG__355	FL-18 CD14-		20	yes	yes		yes	yes		yes
	NOG__356	FL-18 CD14-		20	yes	yes		yes	yes		yes
	NOG__357	FL-18		20	yes	yes	yes	yes	yes	yes	yes
	NOG__358	FL-18		20	yes	yes	yes	yes	yes	yes	yes
	NOG__359	FL-18		15	yes	yes		yes	yes		yes
	NOG__360	FL-18		15	yes	yes		yes	yes		yes
	NOG__361	FL-18		15	yes	yes		yes	yes		yes
	NOG__362	FL-18	found dead, unspecified		*excluded*						
	NOG__363	CB-99		15	yes	yes					
	NOG__364	CB-99		20	yes	yes	yes		yes	yes	
	NOG__365	CB-99		15	yes	yes					
	NOG__366	CB-99		20	yes	yes	yes		yes	yes	
	NOG__367	CB-99		15	yes	*no plasma*					
	NOG__368	CB-99		20	yes	yes	yes		yes	yes	
	NOG__369	CB-99		20	yes	yes	yes		yes	yes	
2	NOG__425	FL-13	found dead, cage flood	12	*up until week 12*						
	NOG__426	FL-13		20	yes	yes	yes		yes	yes	
	NOG__427	FL-13		20	yes	yes	yes		yes	yes	
	NOG__428	FL-13	found dead, cage flood	12	*up until week 12*						
	NOG__436	CB-93		20	yes	yes	yes		yes	yes	
	NOG__437	CB-93		20	yes	yes	yes		yes	yes	
	NOG__438	CB-93		20	yes	yes	yes		yes	yes	
	NOG__439	CB-93		20	yes	yes	yes		yes	yes	
3	NOG__410	FL-15		20	yes	yes	yes		yes	yes	
	NOG__411	FL-15		20	yes	yes	yes		yes	yes	
	NOG__412	FL-15		20	yes	yes	yes		yes	yes	
	NOG__413	FL-15		20	yes	yes	yes		yes	yes	
	NOG__421	CB-111		20	yes	yes	yes		yes	yes	
	NOG__422	CB-111		20	yes	yes	yes		yes	yes	
	NOG__423	CB-111		20	yes	yes	yes		yes	yes	
	NOG__424	CB-111		20	yes	yes	yes		yes	yes	

Table S4. Details of experimental mice used for the engraftment and growth monitoring of A375 melanoma CDX.

Cohort/ Independent experiment	mouse ID	Strain	donor ID	Experimental animal death	endpoint (weeks post-transplant)	Tumor growth/rejection	Adverse effect observations	Analyzed for figures:	Luminex Fig. 5F	Luminex Fig. S9B	Adverse effects FL-4
1	A24 054	NOG-A24 Tg	CB-69	endpoint	26.0	keep growing, no regression	nil	Fig. 5A-B, Fig. S9D			
	A24 055	NOG-A24 Tg	CB-69	endpoint	26.0	keep growing, no regression	nil				
	A24 056	NOG-A24 Tg	CB-69	endpoint	26.0	keep growing, no regression	nil				
	A24 061	NOG-A24 Tg	CB-69	euthanasia due to necrotic tumor	25.4	keep growing, no regression	necrotic tumor				
	A24 062	NOG-A24 Tg	CB-69	euthanasia due to necrotic tumor	25.4	keep growing, no regression	necrotic tumor				
	A24 063	NOG-A24 Tg	CB-69	endpoint	26.1	keep growing, no regression	nil				
	A24 066	NOG-A24 Tg	CB-69	endpoint	26.1	keep growing, no regression	nil				
	A24 067	NOG-A24 Tg	CB-69	endpoint	26.1	palpable (<100mm3), completely rejected	nil				
2	A24 072	NOG-A24 Tg	FL-4	euthanasia due to weakness	23.1	palpable (<100mm3), completely rejected	severe anemia				Fig. S9C
	A24 073	NOG-A24 Tg	FL-4	euthanasia due to weakness	22.6	keep growing, no regression	severe anemia				
	A24 074	NOG-A24 Tg	FL-4	endpoint	23.1	palpable (<100mm3), completely rejected	nil				
	A24 075	NOG-A24 Tg	FL-4	endpoint	23.1	palpable (<100mm3), completely rejected	nil				
	A24 076	NOG-A24 Tg	FL-4	endpoint	23.1	palpable (<100mm3), completely rejected	nil				
	A24 077	NOG-A24 Tg	FL-4	euthanasia due to weakness	22.6	keep growing, no regression	severe anemia				
	A24 078	NOG-A24 Tg	FL-4	endpoint	23.1	keep growing, no regression	nil				
	A24 079	NOG-A24 Tg	FL-4	euthanasia due to weakness	22.6	keep growing, no regression	severe anemia				
	A24 080	NOG-A24 Tg	FL-4	endpoint	23.1	palpable (<100mm3), completely rejected	nil				
	A24 081	NOG-A24 Tg	FL-4	euthanasia due to weakness	23.1	keep growing, no regression	severe anemia				
3	A24 095	NOG-A24 Tg	FL-4	endpoint	24.6	keep growing, no regression	nil				
	A24 100	NOG-A24 Tg	FL-4	euthanasia due to weakness	24.6	keep growing, no regression	severe anemia				
	A24 101	NOG-A24 Tg	FL-4	endpoint	24.7	palpable (<100mm3), completely rejected	nil				
	A24 102	NOG-A24 Tg	FL-4	euthanasia due to weakness	24.6	keep growing, no regression	severe anemia				
	A24 103	NOG-A24 Tg	FL-4	found dead	23.9	palpable (<100mm3), completely rejected	severe anemia				
	A24 104	NOG-A24 Tg	FL-4	endpoint	24.7	palpable (<100mm3), completely rejected	nil				
	A24 107	NOG-A24 Tg	FL-4	endpoint	24.7	palpable (<100mm3), completely rejected	nil				
	A24 110	NOG-A24 Tg	FL-4	euthanasia due to weakness	24.7	palpable (<100mm3), completely rejected	severe anemia				
	A24 111	NOG-A24 Tg	FL-4	endpoint	24.7	palpable (<100mm3), completely rejected	nil				
	A24 112	NOG-A24 Tg	FL-4	euthanasia due to weakness	24.6	regression after day 49	severe anemia				
	A24 113	NOG-A24 Tg	FL-4	endpoint	24.6	keep growing, no regression	nil				
3	A24 094	NOG	FL-4	endpoint	24.7	palpable (<100mm3), completely rejected	nil	Fig. 5C-D, Fig S9D	yes	yes	
	A24 097	NOG	FL-4	endpoint	24.7	palpable (<100mm3), completely rejected	nil		yes	yes	
	A24 098	NOG	FL-4	endpoint	24.6	regression after day 46	slight anemia		yes		
	A24 099	NOG	FL-4	endpoint	24.7	palpable (<100mm3), completely rejected	nil		yes	yes	
	A24 106	NOG	FL-4	endpoint	24.7	palpable (<100mm3), completely rejected	nil		yes	yes	
	A24 108	NOG	FL-4	endpoint	24.7	palpable (<100mm3), completely rejected	nil		yes	yes	
	A24 109	NOG	FL-4	euthanasia due to weakness	24.6	regression after day 42	severe anemia		yes		
	A24 114	NOG	FL-4	endpoint	24.7	palpable (<100mm3), completely rejected	nil		yes	yes	
	A24 115	NOG	FL-4	endpoint	24.7	palpable (<100mm3), completely rejected	nil		yes	yes	
	A11 76	NOG	FL-4	endpoint	24.7	palpable (<100mm3), completely rejected	nil				
4	NOG 84	NOG	FL-4	endpoint	23.1	palpable (<100mm3), completely rejected	nil				
	NOG 85	NOG	FL-4	endpoint	23.1	palpable (<100mm3), completely rejected	nil				
	NOG 86	NOG	FL-4	endpoint	23.1	keep growing, no regression	nil				
	NOG 87	NOG	FL-4	endpoint	23.1	palpable (<100mm3), completely rejected	nil				
	NOG 88	NOG	FL-4	endpoint	23.1	keep growing, no regression	nil				
	NOG 89	NOG	FL-4	endpoint	23.1	keep growing, no regression	nil				
	NOG 90	NOG	FL-4	endpoint	23.1	keep growing, no regression	slight anemia				
	NOG 91	NOG	FL-4	euthanasia due to weakness	21.1	palpable (<100mm3), no regression	severe anemia				
	NOG 92	NOG	FL-4	endpoint	23.1	palpable (<100mm3), completely rejected	nil				
	NOG 93	NOG	FL-4	endpoint	23.1	keep growing, no regression	nil				
3	A11 050	NOG	FL-13	endpoint	24.7	palpable (<100mm3), completely rejected	nil				
	A11 051	NOG	FL-13	endpoint	24.6	keep growing, no regression	nil		yes		
	A11 052	NOG	FL-13	endpoint	24.7	palpable (<100mm3), completely rejected	nil		yes	yes	
	A11 053	NOG	FL-13	endpoint	24.7	palpable (<100mm3), completely rejected	nil		yes	yes	
	A11 054	NOG	FL-13	endpoint	24.6	keep growing, no regression	nil		yes		
	A11 057	NOG	FL-13	endpoint	24.7	palpable (<100mm3), completely rejected	nil				
	A11 058	NOG	FL-13	endpoint	24.7	palpable (<100mm3), completely rejected	nil		yes	yes	
	A11 062	NOG	FL-13	endpoint	24.7	palpable (<100mm3), completely rejected	nil		yes	yes	
	A11 067	NOG	FL-13	endpoint	24.7	keep growing, no regression	nil				
	A11 071	NOG	FL-13	endpoint	24.7	palpable (<100mm3), completely rejected	nil				
	A11 073	NOG	FL-13	endpoint	24.6	keep growing, no regression	nil		yes		
	A11 074	NOG	FL-13	endpoint	24.6	keep growing, no regression	nil				
	A11 075	NOG	FL-13	endpoint	24.6	keep growing, no regression	watery stool		yes		

Table S5. Antibodies for assessing the kinetics of human immune cell reconstitution in peripheral blood

Antigen	Fluorochrome	Reactivity	Clone	Supplier	Catalog Number
CD45	APC	anti-mouse	30-F11	Biolegend	103112
CD45	APC-Cy7	anti-human	2D1	Biolegend	368516
CD3	PE-Cy7	anti-human	HIT3a	Biolegend	300316
CD19	PE	anti-human	HIB19	Biolegend	302208
CD33	FITC	anti-human	HIM3-4	Biolegend	303304

Table S6. Antibody panels for the analysis of humanized mice tissues

Endpoint tissue analysis panel					
Antigen	Fluorochrome	Reactivity	Clone	Supplier	Catalog Number
CD45	PerCP-Cy5.5	anti-mouse	30-F11	BD	550994
CD56	FITC	anti-human	REA196	Miltenyi	130-114-740
CD11b	PE-Cy7	anti-mouse/human	M1/70	Biolegend	101216
CD11c	PE/Dazzle594	anti-human	3.9	Biolegend	301640
CD68	PE	anti-human	Y1/82A	Biolegend	333808
HLA-DR	BV785	anti-human	L243	Biolegend	307642
CD8a	BV711	anti-human	RPA-T8	Biolegend	301044
CD19	BV650	anti-human	SJ25C1	BD	563226
CD16	BV605	anti-human	3G8	BD	563172
CD45	BV510	anti-human	HI30	Biolegend	304036
GR-1	BV421	anti-mouse	rb6-8c5	Biolegend	108433
CD66b	BV421	anti-human	G10F5	BD	562940
CD4	APC-Cy7	anti-human	RPA-T4	BD	557871
F4/80	APC	anti-mouse	BM8	Biolegend	123116
CD3	APC	anti-human	UCHT1	Biolegend	300412
CD14	BUV737	anti-human	M5E2	BD	612763
CD33	BUV395	anti-human	WM53	BD	740293
Endpoint lung analysis panel					
Antigen	Fluorochrome	Reactivity	Clone	Supplier	Catalog Number
FcεRIα	PerCP	anti-human	AER-37	Biolegend	334616
CD88	PE-Cy7	anti-human	S5/1	Biolegend	344308
CD66b	PE/Dazzle594	anti-human	G10F5	Biolegend	305122
Siglec-1	PE	anti-human	7-239	BD	565248
HLA-DR	BV785	anti-human	L243	Biolegend	307642
CD3	BV650	anti-human	SP34-2	BD	563916
CD19	BV650	anti-human	SJ25C1	BD	563226
CD20	BV650	anti-human	2H7	BD	563780
CD163	BV605	anti-human	GHI/61	Biolegend	333616
CD45	V500	anti-human	HI30	BD	560777
CD1c	BV421	anti-human	L161	Biolegend	331526
CD16	APC-Cy7	anti-human	3G8	Biolegend	302018
CD14	Alexa Fluor 700	anti-human	M5E2	BD	557923
CD45	BUV737	anti-mouse	30-F11	BD	748371
CD123	BUV395	anti-human	7G3	BD	564195
unlabeled	CADM1	anti-human	9D2	MBL Life Science	CM005-3
IgY	Alexa Fluor 647	anti-chicken	Polyclonal	Jackson Immunoresearch	703-606-155

Table S7. Correlation of chimerism and tissue cell numbers with liver pathology

	CB $CD34^+$				FL $CD34^+$			
		Sperman r	P (two-tailed)	P value summary		Sperman r	P (two-tailed)	P value summary
BM	%hCD45	0.645	0.0273	*	%hCD45	0.157	0.7109	ns
	$CD66b^+$ progenitors	-0.186	0.5583	ns	$CD66b^+$ progenitors	-0.566	0.1506	ns
	imm Neu #	0.079	0.8078	ns	imm Neu #	-0.482	0.2325	ns
	mat Neu #	0.057	0.8608	ns	mat Neu #	-0.723	0.0512	ns
	$CD14^+$Mo #	-0.398	0.1991	ns	$CD14^+$Mo #	-0.8434	0.0127	*
	$CD16^+$Mo #	0.219	0.4908	ns	$CD16^+$Mo #	0.542	0.1706	ns
	$CD11c^+$DC #	0.126	0.6956	ns	$CD11c^+$DC #	0.108	0.7972	ns
	$AutoF^+$ Macs	-0.298	0.3440	ns	$AutoF^+$ Macs	0.410	0.3127	ns
	$HLADR^-$ Macs #	-0.050	0.8788	ns	$HLADR^-$ Macs #	-0.193	0.6500	ns
	$HLADR^+$ Macs #	-0.161	0.6134	ns	$HLADR^+$ Macs #	-0.590	0.1302	ns
	B #	-0.420	0.1744	ns	B #	-0.819	0.0187	*
	$CD4^+$ T #	0.613	0.0380	*	$CD4^+$ T #	0.771	0.0323	*
	$CD8^+$ T #	0.645	0.0273	*	$CD8^+$ T #	0.639	0.0960	ns
	NK #	0.165	0.6055	ns	NK #	0.603	0.1228	ns
BLOOD	%hCD45	0.420	0.1744	ns	%hCD45	0.747	0.0411	*
	Neu #	-0.358	0.5000	ns	Neu #	0.417	0.5000	ns
	$CD14^+$Mo #	-0.739	0.0083	**	$CD14^+$Mo #	0.747	0.0411	*
	$CD16^+$Mo #	-0.643	0.0277	*	$CD16^+$Mo #	0.916	0.0034	**
	$CD11c^+$DC #	-0.445	0.1481	ns	$CD11c^+$DC #	0.217	0.6032	ns
	B #	-0.825	0.0017	**	B #	-0.482	0.2325	ns
	$CD4^+$ T #	0.452	0.1412	ns	$CD4^+$ T #	0.892	0.0054	**
	$CD8^+$ T #	0.412	0.1823	ns	$CD8^+$ T #	0.819	0.0187	*
	NK #	0.308	0.3265	ns	NK #	0.873	0.0068	**
SPLEEN	%hCD45	0.441	0.1516	ns	%hCD45	0.723	0.0512	ns
	Neu #	0.073	0.8212	ns	Neu #	0.554	0.1621	ns
	$CD14^+$Mo #	-0.258	0.4142	ns	$CD14^+$Mo #	0.988	0.0002	***
	$CD16^+$Mo #	0.054	0.8696	ns	$CD16^+$Mo #	0.964	0.0008	***
	$CD11c^+$DC #	0.186	0.5583	ns	$CD11c^+$DC #	0.892	0.0054	**
	$HLADR^-$ Macs #	0.437	0.1554	ns	$HLADR^-$ Macs #	0.603	0.1228	ns
	$HLADR^+$ Macs #	-0.100	0.7555	ns	$HLADR^+$ Macs #	0.807	0.0208	*
	B #	0.377	0.2262	ns	B #	0.241	0.5647	ns
	$CD4^+$ T #	0.746	0.0074	**	$CD4^+$ T #	0.843	0.0127	*
	$CD8^+$T #	0.778	0.0042	**	$CD8^+$T #	0.723	0.0512	ns
	NK #	0.710	0.0125	*	NK #	0.807	0.0208	*
LIVER	%hCD45	0.550	0.0837	ns	%hCD45	0.883	0.0151	*
	Neu #	0.070	0.8378	ns	Neu #	-0.018	0.9881	ns
	$CD11c^+$DC #	-0.202	0.5479	ns	$CD11c^+$DC #	-0.541	0.2183	ns
	$CD11c^{high}$ DC #	0.136	0.6874	ns	$CD11c^{high}$ DC #	0.775	0.0508	ns
	$CD14^{high}$ Mac #	0.094	0.7838	ns	$CD14^{high}$ Mac #	0.829	0.0302	*
	B #	0.343	0.2990	ns	B #	-0.072	0.8873	ns
	$CD4^+$ T #	0.587	0.0621	ns	$CD4^+$ T #	0.901	0.0095	**
	$CD8^+$ T #	0.667	0.0297	*	$CD8^+$ T #	0.721	0.0778	ns
	NK #	0.639	0.0394	*	NK #	0.955	0.0040	**
LUNG	%hCD45	0.645	0.0273	*	%hCD45	0.699	0.0627	ns
	Neu #	-0.262	0.4074	ns	Neu #	0.133	0.7565	ns
	$CD14^+$Mo #	-0.688	0.0165	*	$CD14^+$Mo #	0.747	0.0411	*
	$CD16^+$ Mo #	-0.215	0.4982	ns	$CD16^+$ Mo #	0.868	0.0091	**
	$CD14^+CD16^+$ iMo #	-0.642	0.0282	*	$CD14^+CD16^+$ iMo #	0.819	0.0187	*
	$CD163^+$ Macs #	-0.022	0.9506	ns	$CD163^+$ Macs #	0.711	0.0565	ns
	$CD163^-$ Macs #	0.380	0.2216	ns	$CD163^-$ Macs #	0.892	0.0054	**
	pDC #	0.226	0.4763	ns	pDC #	0.639	0.0960	ns
	preDC #	0.047	0.8875	ns	preDC #	0.506	0.2056	ns
	cDC1 #	0.022	0.9506	ns	cDC1 #	0.771	0.0323	*
	cDC2 #	-0.545	0.0703	ns	cDC2 #	0.795	0.0244	*

Table S8. Correlation of circulatory inflammatory mediators with liver pathology

		CB CD34+			FL CD34+		
		Sperman r	P (two-tailed)	P value summary	Sperman r	P (two-tailed)	P value summary
Luminex (pg/mL)	EGF	0.292	0.3524	ns	0.050	0.8860	ns
	FGF-2	0.614	0.0376	*	-0.267	0.4228	ns
	Flt-3L	0.851	0.0008	***	0.718	0.0156	*
	Fractalkine	0.182	0.5678	ns	0.439	0.1759	ns
	G-CSF	0.036	0.9137	ns	-0.016	0.9645	ns
	GM-CSF	0.771	0.0048	**	0.720	0.0154	*
	GRO (CXCL1)	0.133	0.6789	ns	0.447	0.1684	ns
	IFNy	0.713	0.0118	*	0.703	0.0190	*
	IL-10	0.635	0.0305	*	0.746	0.0107	ns
	IL-12P40	0.420	0.1726	ns	0.837	0.0024	**
	IL-12P70	0.731	0.0091	**	0.725	0.0147	*
	IL-13	0.584	0.0498	*	0.627	0.0432	*
	IL-15	0.778	0.0042	**	0.890	0.0005	**
	IL-17A	0.688	0.0165	*	0.576	0.0682	**
	IL-1RA	0.820	0.0018	**	0.668	0.0285	*
	IL-4	0.313	0.3176	ns	0.482	0.1349	ns
	IL-5	0.618	0.0359	*	0.871	0.0011	**
	IL-6	0.853	0.0003	***	0.790	0.0010	**
	IL-7	-0.025	0.9381	ns	0.048	0.8887	ns
	IL-8	0.782	0.0040	**	0.886	0.0006	**
	IL-9	0.696	0.0151	*	0.741	0.0114	*
	IP-10 (CXCL10)	0.570	0.0567	ns	0.860	0.0012	**
	MCP-1 (CCL2)	0.692	0.0157	*	0.977	3.81E-06	****
	MCP-3 (CCL7)	0.601	0.0423	*	0.520	0.1033	ns
	MDC (CCL22)	0.430	0.1628	ns	0.717	0.0162	*
	MIP-1a (CCL3)	0.708	0.0134	*	0.814	0.0040	**
	MIP-1b (CCL4)	0.726	0.0103	*	0.825	0.0025	**
	RANTES	-0.581	0.0516	ns	0.370	0.2612	ns
	TNFa	0.667	0.0214	*	0.774	0.0071	**
	VEGF	0.133	0.6765	ns	-0.002	0.9973	ns
	sCD40L	-0.199	0.5290	ns	0.247	0.4617	ns
ELISA (ng/mL)	ALT	0.674	0.0197	*	0.338	0.3071	ns

Table S9. HLA typing results.

Tumor cell line		
Cell line	Allele I	Allele II
A375	A*01:01	A*02:01
$CD34^+$ HSPC		
Donor	Allele I	Allele II
KKH69	A*24:02	A*24:07
FL-4	A*11:01	A*24:02
FL-13	A*11:01	A*34:01
